# Chilling-induced phosphorylation of IPA1 by OsSAPK6 activates chilling tolerance responses in rice

**DOI:** 10.1038/s41421-022-00413-2

**Published:** 2022-07-26

**Authors:** Meiru Jia, Xiangbing Meng, Xiaoguang Song, Dahan Zhang, Liquan Kou, Junhui Zhang, Yanhui Jing, Guifu Liu, Huihui Liu, Xiahe Huang, Yingchun Wang, Hong Yu, Jiayang Li

**Affiliations:** 1 State Key Laboratory of Plant Genomics and National Center for Plant Gene Research, Institute of Genetics and Developmental Biology, Innovation Academy for Seed Design, Chinese Academy of Sciences, Beijing, China; 2 University of Chinese Academy of Sciences, Beijing, China; 3 State Key Laboratory of Molecular Developmental Biology, Institute of Genetics and Developmental Biology, Innovation Academy for Seed Design, Chinese Academy of Sciences, Beijing, China

**Keywords:** Plant signalling, Plant molecular biology

## Abstract

Chilling is a major abiotic stress harming rice development and productivity. The C-REPEAT BINDING FACTOR (CBF)-dependent transcriptional regulatory pathway plays a central role in cold stress and acclimation in *Arabidopsis*. In rice, several genes have been reported in conferring chilling tolerance, however, the chilling signaling in rice remains largely unknown. Here, we report the chilling-induced OSMOTIC STRESS/ABA-ACTIVATED PROTEIN KINASE 6 (OsSAPK6)-IDEAL PLANT ARCHITECTURE 1 (IPA1)-OsCBF3 signal pathway in rice. Under chilling stress, OsSAPK6 could phosphorylate IPA1 and increase its stability. In turn, IPA1 could directly bind to the GTAC motif on the *OsCBF3* promoter to elevate its expression. Genetic evidence showed that OsSAPK6, IPA1 and OsCBF3 were all positive regulators of rice chilling tolerance. The function of OsSAPK6 in chilling tolerance depended on IPA1, and overexpression of *OsCBF3* could rescue the chilling-sensitive phenotype of *ipa1* loss-of-function mutant. Moreover, the natural gain-of-function allele *ipa1-2D* could simultaneously enhance seedling chilling tolerance and increase grain yield. Taken together, our results revealed a chilling-induced OsSAPK6-IPA1-OsCBF signal cascade in rice, which shed new lights on chilling stress-tolerant rice breeding.

## Introduction

Cold is a major abiotic stress that adversely affects plant growth and crop production^1,2^. Rice, a staple food feeding half of the world population, is a chilling sensitive plant that shows slow seedling growth, yellowing, stunting, withering, and ultimately death under low temperature stress^3,4^. Therefore, it is crucially needed to develop genetic tools for breeding cold stress-tolerant rice varieties, which can prevent low temperature damage and minimalize the constraint of rice cultivation area^5,6^.

Cold response has been extensively studied in *Arabidopsis*, and the C-REPEAT BINDING FACTOR/DEHYDRATION-RESPONSIVE ELEMENT-BINDING PROTEIN 1 (CBF/DREB1)-dependent transcriptional regulatory pathway plays a central role in cold stress and acclimation^1,7^. The expressions of three *CBF* genes are rapidly induced under cold stress, and CBF proteins could directly bind to the CRT/DRE cis elements in the promoters of *COLD REGULATED* (*COR*) genes, leading to enhanced cold tolerance in *Arabidopsis*^1,7,8^. INDUCER OF CBF EXPRESSION 1 (ICE1) is the key transcription factor in the upstream of *CBFs* in cold responses, which could directly bind to MYC recognition sequences in the promoter of *CBFs*^9^. Under normal conditions, ICE1 is degraded by the E3 ligase HIGH EXPRESSION OF OSMOTICALLY RESPONSIVE GENE 1 (HOS1), but under cold stress, ICE1 is phosphorylated by OPEN STOMATA 1 (OST1)/SUCROSE NON-FERMENTING 1 (SNF1)-related protein kinase 2.6 (SnRK2.6) for inhibiting its degradation, which in turn positively regulates *CBF* expression to enhance cold tolerance^10,11^.

Although the ICE1-CBFs-COR cold signaling cascade has been well-characterized for *Arabidopsis*, the understandings of cold tolerance for rice are fragmental. OsICE1/ BASIC HELIX-LOOP-HELIX PROTEIN 002 (OsbHLH002) could be phosphorylated and stabilized by MITOGEN-ACTIVATED PROTEIN KINASE 3 (OsMAPK3) to increase *TREHALOSE-6-PHOSPHATE PHOSPHATASE 1* (*OsTPP1*) expression and enhance the chilling tolerance^12^. Cyclic nucleotide-gated channel 9 (OsCNGC9), a cyclic nucleotide-gated channel, could positively regulate rice chilling tolerance by mediating Ca^2+^ elevation in cytosol^13^. Under chilling stress, OsCNGC9 could be phosphorylated and stabilized by OSMOTIC STRESS/ABA-ACTIVATED PROTEIN KINASE 8 (OsSAPK8), the closest rice homolog of OST1. The expression of *OsCBF3*/*OsDREB1A* is cold induced and *oscbf3* mutant is chilling sensitive. OsCBF3 could directly bind to the DRE-box motif on the promoter of *OsCNGC9* to upregulate its expression under chilling stress^13^. CHILLING TOLERANCE DIVERGENCE 1 (COLD1), different alleles of which conferred the chilling tolerance variation between *indica* and *japonica* species, could work with G-PROTEIN α SUBUNIT 1 (RGA1) to mediate the cold-induced Ca^2+^ influx to promote cold stress, and may function as a chilling sensor in rice^14–16^. Overexpression of *CBL-INTERACTING PROTEIN KINASE 7* (*OsCIPK7*) could increase the chilling tolerance through Ca^2+^ influx^17^. The Ca^2+^ influx and the protein kinases have been revealed to play an important role in rice chilling sensing and tolerance. However, the cold signal cascade, especially the regulatory mechanisms underlying cold-induced *CBF* expression, was unclear in rice.

In plants, the activation of defense responses under biotic or abiotic stresses usually inhibit their own growth, and the genetic resources or tools that can enhance the resistance without harming crop yield are long desired^5,8,18^. *IDEAL PLANT ARCHITECTURE 1* (*IPA1*)/ *WEALTHY FARMER’S PANICLE* (*WFP*)/*OsSPL14* gene, encoding a SQUAMOSA promoter-binding protein-like (SPL) transcription factor, has been reported as a critical factor regulated by miRNA156/529 in shaping rice ideal plant architecture and substantially increasing rice grain yield^19–22^. *IPA1* could also positively regulate disease resistance against *Magnaporthe oryzae* and bacterial blight caused by *Xanthomonas oryzae pv. oryzae* (*Xoo*), conferring a promotion of both yield and disease resistance by sustaining a balance between growth and immunity^23–25^. Similar resources have been long-desired for abiotic stresses, and elucidating the mechanism underlying the tradeoff between growth and chilling tolerance will greatly benefit rice breeding.

In this study, we screened out and identified a chilling-sensitive mutant *ossapk6*. Biochemical analyses revealed that OsSAPK6 could phosphorylate IPA1 and stabilize IPA1 under chilling stress. IPA1 could directly bind to the *OsCBF3* promoter via the GTAC motif and induce *OsCBF3* expression. Overexpression of *OsSAPK6*, *IPA1*, and *OsCBF3* could each enhance chilling tolerance, and gain-of-function allele *ipa1-2D* promoted both rice yield and seedling chilling tolerance. Our study has revealed an OsSAPK6-IPA1-OsCBF chilling signaling cascade in rice, which has greatly expanded our understanding of chilling responses and provided important genetic resources for breeding high yield and chilling-tolerant rice varieties.

## Results

### OsSAPK6 positively regulates chilling tolerance in rice

**Fig. 1:**
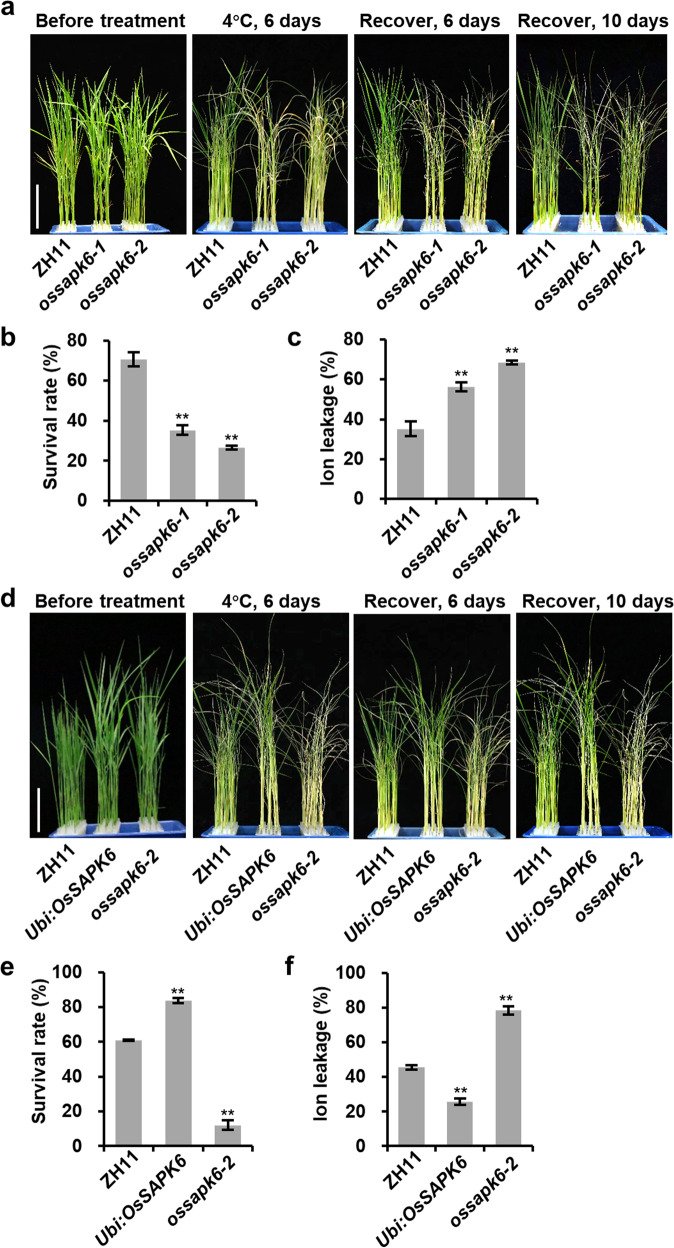
OsSAPK6 positively regulates chilling tolerance in rice. **a** Plant morphologies of 2-week-old wild-type ZH11, *ossapk6-1* and *ossapk6-2* seedlings before treatment, after 4 °C treatment for 6 days, and subsequent recovery for 6 or 10 days. **b** Survival rates of ZH11, *ossapk6-1,* and *ossapk6-2* after 4 °C treatment as shown in **a**. **c** Ion leakage levels of ZH11, *ossapk6-1* and *ossapk6-2* after 4 °C treatment for 6 h. **d** Plant morphologies of 2-week-old ZH11, *Ubi:OsSAPK6* and *ossapk6-2* seedlings before treatment, after 4 °C treatment for 6 days, and subsequent recovery for 6 or 10 days. **e** Survival rates of ZH11, *Ubi:OsSAPK6* and *ossapk6-2* after 4 °C treatment as shown in **d**. **f** Ion leakage levels of ZH11, *Ubi:OsSAPK6* and *ossapk6-2* after 4 °C treatment for 6 h. In **b**, **c**, **e**, and **f**, values are means ± SD (*n* = 3 biological replicates), and the asterisks indicate significant differences compared with the ZH11 (**P < 0.01, Student’s *t-*test). Bars = 5 cm in **a** and **d**. See also Supplementary Figs. S1 and S2.

From the rice mutant library generated by CRISPR-Cas9 in the ZH11 (Zhong Hua 11) background^26^, we found one chilling sensitive mutant LBM3705. By sequencing the guide sequence and its target gene in LBM3705, we found one “A” insertion at the 42-bp site in exon 1 of *OsSAPK6*, which resulted in a frameshift and premature translation termination of *OsSAPK6* (Supplementary Fig. S1). We therefore named this mutant as *ossapk6-1*. After six-day chilling treatment, the leaves of 2-week-old *ossapk6-1* seedlings turned yellow (Fig. 1a), and the survival rate of *ossapk6-1* had significantly decreased to 35.3% compared with 70.6% of ZH11 (Fig. 1b). We then examined the ion leakage, an indicator of plasma membrane damage induced by cold stress, and found that the ion leakage in *ossapk6-1* was significantly higher than that in ZH11 after ten-hour chilling treatment (Fig. 1c). Another *OsSAPK6* mutant line, *ossapk6-2*, which harbored one “T” insertion at the 42-bp site in exon 1 (Supplementary Fig. S1), exhibited similar chilling sensitive phenotype and cold-induced elevation of ion leakage to *ossapk6-1* (Fig. 1a–c). To further confirm this result, we generated overexpression transgenic lines of *OsSAPK6* driven by the rice *Ubiquitin* promoter (*Ubi:OsSAPK6*) in ZH11 background. Under normal conditions, 2-week-old seedlings of *Ubi:OsSAPK6* was significantly taller than ZH11 (Supplementary Fig. S2a). Under chilling stresses, *Ubi:OsSAPK6* line showed enhanced chilling tolerance with increased survival rate and decreased ion leakage compared with ZH11 (Fig. 1d–f; Supplementary Fig. S2b). All these results demonstrated that OsSAPK6 was a positive regulator of rice chilling tolerance.

### OsSAPK6 phosphorylates and stabilizes IPA1

**Fig. 2:**
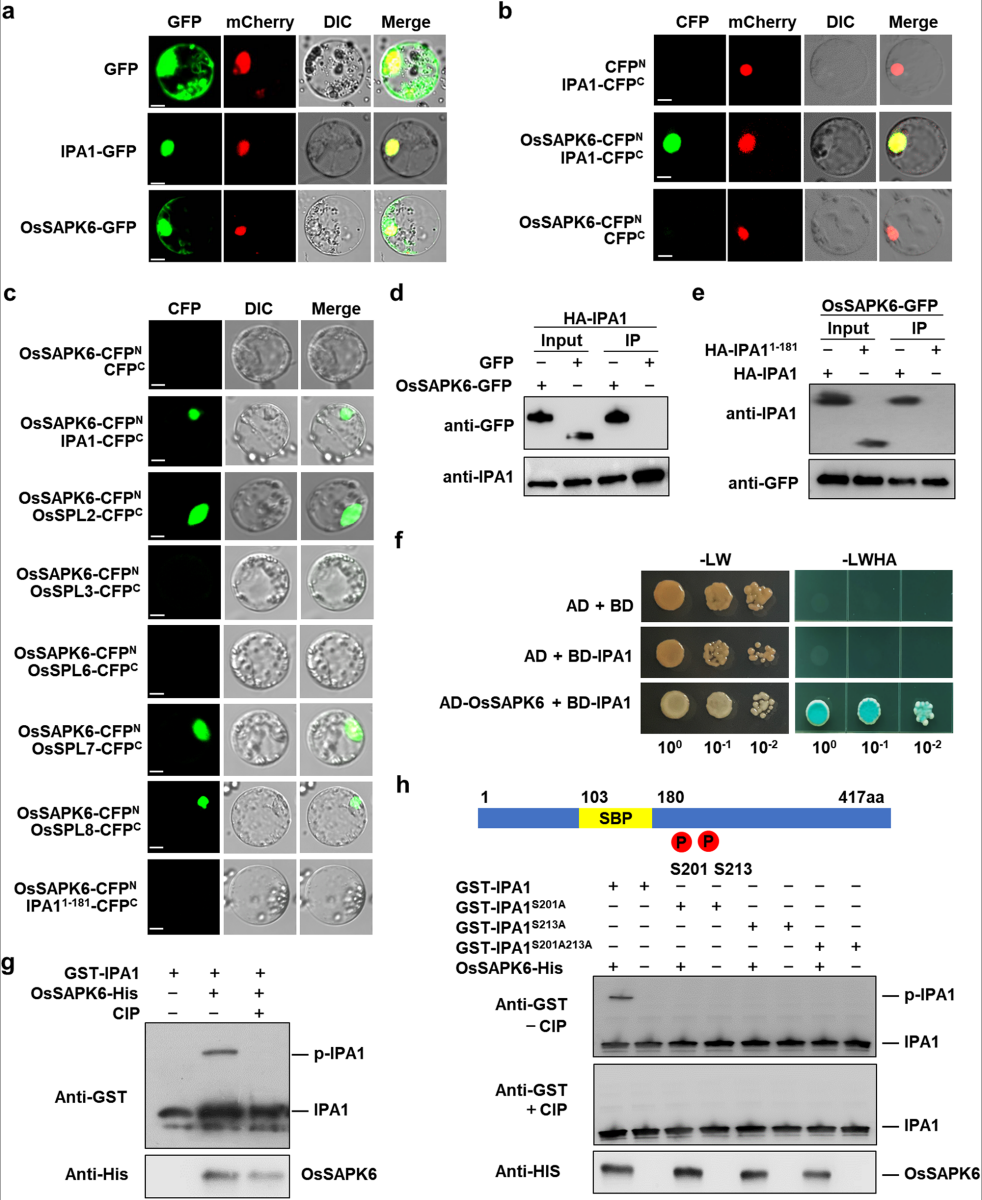
OsSAPK6 physically interacts with and phosphorylates IPA1. **a** Subcellular localization of IPA1 and OsSAPK6 in rice protoplasts. Scale bars, 5 µm. **b** BiFC showing the interaction between IPA1 and OsSAPK6 in rice protoplasts. Scale bars, 5 µm. **c** BiFC showing the interaction between OsSPLs and OsSAPK6 in rice protoplasts. Scale bars, 10 µm. **d** Co-IP assays demonstrating the interaction between IPA1 and OsSAPK6. **e** HA-IPA1 but not HA-IPA1^1–181^ could be co-immunoprecipitated when OsSAPK6-GFP was pulled down from protoplast extracts. **f** Yeast two-hybrid assays demonstrating the interaction between IPA1 and OsSAPK6. **g** IPA1 phosphorylation mediated by OsSAPK6 in vitro. The purified recombinant OsSAPK6-His and GST-IPA1 were incubated in protein kinase buffer and separated on 12% (w/v) SDS-PAGE containing 50 µM Phos-tag. **h** Detection of phosphorylation of GST-IPA1, GST-IPA1^S201A^, GST-IPA1^S213A^, and GST-IPA1^S201A213A^ by OsSAPK6 in vitro. Recombinant proteins were incubated with OsSAPK6, separated in a Phos-tag gel, and detected with anti-GST monoclonal antibody. See also Supplementary Fig. S3 and Table S1.

OsSAPK6 belongs to the SnRK2 family^27^. To identify the substrates of OsSAPK6, we performed co-immunoprecipitation (co-IP) followed by mass spectrometry using the GFP-OsSAPK6 fusion protein transiently expressed in rice protoplasts. The result showed that IPA1 was highly enriched in candidate interacting proteins (Supplementary Fig. S3a and Table S1). As IPA1 is a transcription factor localized in nucleus, we first examined the subcellular localization of OsSAPK6. We transformed *35* *S:SAPK6-GFP* into rice leaf protoplasts with nucleus marker *35S:mCherry-NLS*, and observed that OsSAPK6-GFP was localized in both the nucleus and the cytoplasm (Fig. 2a). To test whether OsSAPK6 could truly interact with IPA1, we first conducted bimolecular fluorescence complementation (BiFC) assays, and found cyan fluorescent protein (CFP) fluorescence signal at the nucleus of rice leaf protoplasts when co-expressing 35S:OsSAPK6-CFP^N^ with 35S:IPA1-CFP^C^, 35S:OsSPL2-CFP^C^, 35S:OsSPL7-CFP^C^, or 35S:OsSPL8-CFP^C^ but not with 35S:IPA1^1–181^-CFP^C^, 35S:OsSPL3-CFP^C^, or 35S:OsSPL6-CFP^C^ (Fig. 2b, c). Furthermore, we tested their interaction using co-IP and transiently co-expressed IPA1 fused with a HA tag (HA-IPA1) and OsSAPK6 fused with GFP (OsSAPK6-GFP) in rice protoplasts. OsSAPK6-GFP was co-immunoprecipitated when HA-IPA1 was pulled down from protoplast extracts with anti-IPA1 monoclonal antibody (Fig. 2d), and HA-IPA1 but not HA-IPA1^1–181^ could be co-immunoprecipitated when OsSAPK6-GFP was pulled down (Fig. 2e). The interaction between OsSAPK6 and IPA1 was further confirmed by the yeast two-hybrid assay (Fig. 2f). Moreover, we found that the C-terminal region of IPA1 (182–417 aa) was critical for its interaction with OsSAPK6 (Supplementary Fig. S3b), which was consistent with the BiFC results. These results indicate that OsSAPK6 can interact with IPA1.

We then tested whether IPA1 is a substrate of OsSAPK6. The GST-IPA1 and His-OsSAPK6 recombinant proteins were expressed and purified from *Escherichia coli*, which were co-incubated in kinase buffer and detected by the phos-tag mobility shift assay. The result clearly showed that IPA1 could be phosphorylated by OsSAPK6 (Fig. 2g). Previous report showed that the SnRK2 family protein kinase could phosphorylate the conserved motif (RXXpS/T)^28^. We found that Ser201 and Ser213 of the IPA1 protein are within this motif and might be the phosphorylation sites of OsSAPK6. To test this, we mutated Ser201 and Ser213 to alanines to generate IPA1^S201AS213A^ to mimic non-phosphorylated state. Although the S201AS213A mutations did not alter IPA1 nucleus localization and the interaction of IPA1 with OsSAPK6 (Supplementary Fig. S3c, d), the in vitro phosphorylation assay showed that the phosphorylation of IPA1 could not be detected if any of Ser201 and Ser213 was mutated (Fig. 2h). All these results demonstrated that IPA1 was the substrate of OsSAPK6 with Ser201 and Ser213 as the major phosphorylation residues.

**Fig. 3:**
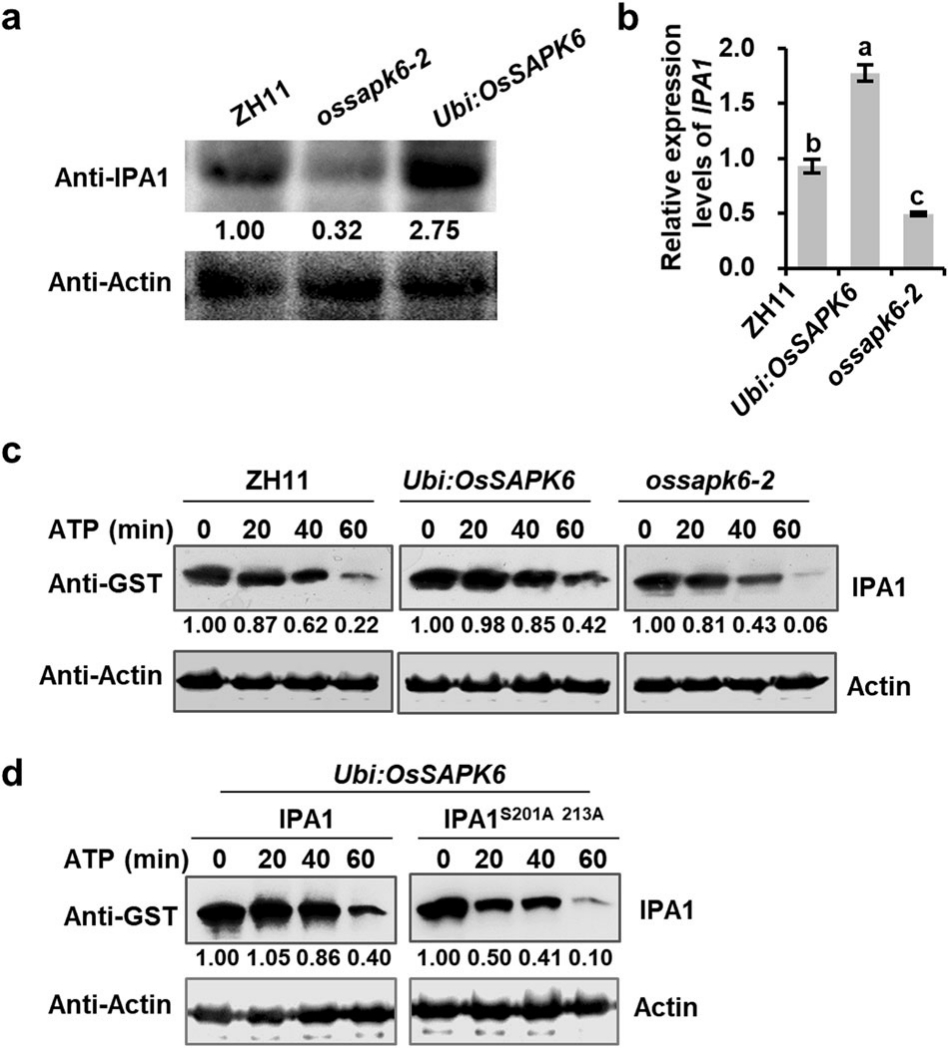
OsSAPK6 enhances the stability of IPA1. **a** Protein levels of IPA1 in ZH11, *Ubi:OsSAPK6* and *ossapk6-2* 2-week-old seedlings. **b** Expression levels of *IPA1* in ZH11, *Ubi:OsSAPK6* and *ossapk6-2* 2-week-old seedlings. Values are means ± SD (*n* = 3 biological replicates) and the different letters indicate significant differences (*P* < 0.01) according to Tukey’s honest significant difference (HSD) test. **c** In vitro cell-free degradation assays showing degradation of GST-IPA1 in extracts from ZH11, *Ubi:OsSAPK6*, and *ossapk6-2* plants in the presence of ATP. **d** In vitro cell-free degradation assay showing degradation of GST-IPA1 and GST-IPA1^S201A213A^ in the extracts from *Ubi:OsSAPK6* plants in the presence of ATP. GST-IPA1 was detected with anti-GST monoclonal antibody and quantitated by densitometry with actin as a control. See also Supplementary Fig. S4.

As phosphorylation may affect protein stability, we first tested the *IPA1* expression levels and protein contents in *OsSAPK6-OE* transgenic plants and *ossapk6-2* mutant, and found that both RNA expression level and the protein level of IPA1 were higher in *OsSAPK6-OE* but lower in *ossapk6-2* compared with those in ZH11 (Fig. 3a, b), suggesting that OsSAPK6 may affect IPA1 at both transcriptional level and post-transcriptional level. We therefore performed a cell-free degradation assay by incubating purified recombinant GST-IPA1 proteins with total proteins extracted from ZH11, *Ubi:OsSAPK6*, or *ossapk6-2* plants. The results showed that GST-IPA1 was degraded more rapidly with *ossapk6-2* than with ZH11 but more slowly with *Ubi:OsSAPK6* extracts (Fig. 3c). Moreover, the non-phosphorylated form of IPA1^S201AS213A^ showed increased degradation rate than IPA1 in cell-free degradation assay using total proteins extracted from *Ubi:OsSAPK6* plants (Fig. 3d; Supplementary Fig. S4). These results demonstrated that OsSAPK6 not only phosphorylates IPA1 to increase its protein stability but also upregulates *IPA1* expression, which may collaboratively lead to the increased IPA1 protein levels.

### The regulation of chilling tolerance by OsSAPK6 is dependent on IPA1

**Fig. 4:**
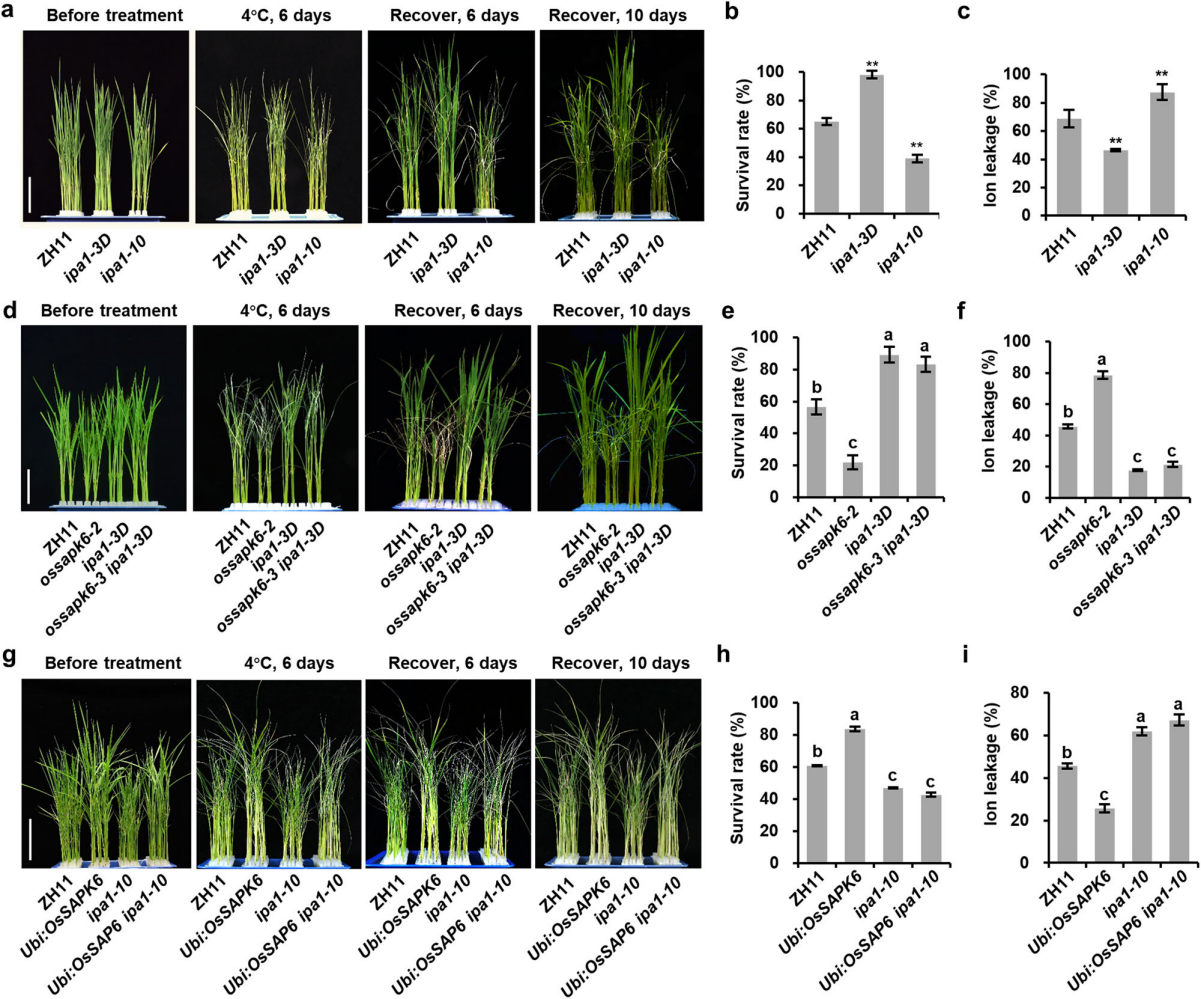
The regulation of chilling tolerance by OsSAPK6 is dependent on IPA1. **a** Plant morphologies of 2-week-old ZH11, *ipa1-3D* and *ipa1-10* seedlings before treatment, after 4 °C treatment for 6 days, and subsequent recovery for 6 or 10 days. **b** Survival rates of ZH11, *ipa1-3D* and *ipa1-10* after 4 °C treatment as shown in **a**. **c** Ion leakage levels of ZH11, *ipa1-3D*, and *ipa1-10* after 4 °C treatment for 6 h. **d** Plant morphologies of 2-week-old ZH11*, ossapk6-2, ipa1-3D*, and *ossapk6-3 ipa1-3D* seedlings before treatment, after 4 °C treatment for 6 days, and subsequent recovery for 6 or 10 days. **e** Survival rates of ZH11*, ossapk6-2*, *ipa1-3D,* and *ossapk6-3 ipa1-3D* after 4 °C treatment as shown in **d**. **f** Ion leakage levels of ZH11, *ossapk6-2*, *ipa1-3D* and *ossapk6-3 ipa1-3D* after 4 °C treatment for 6 h. **g** Plant morphologies of 2-week-old ZH11*, Ubi:OsSAPK6*, *ipa1-10*, and *Ubi:OsSAPK6 ipa1-10* seedlings before treatment, after 4 °C treatment for 6 days, and recovery for 6 or 10 days. **h** Survival rates of ZH11*, Ubi:OsSAPK6*, *ipa1-10,* and *Ubi:OsSAPK6 ipa1-10* after 4 °C treatment as shown in **g**. **i** Ion leakage levels of ZH11, *Ubi:OsSAPK6, ipa1-10*, and *Ubi:OsSAPK6 ipa1-10* after 4 °C treatment for 6 h. Values are means ± SD (*n* = 3 biological replicates). In **b** and **c**, the asterisks indicate significant differences compared with ZH11 (**P < 0.01, Student’s *t*-test). In **e**, **f**, **h**, and **i**, the different letters indicated significant differences (*P* < 0.01) according to Tukey’s HSD test. Bars = 5 cm in **a**, **d** and **g**. See also Supplementary Fig. S5.

To test whether IPA1 functioned in the downstream of OsSAPK6 in cold responses, we first tested the chilling tolerance of the gain-of-function mutant *ipa1-3D* and loss-of-function mutant *ipa1-10*^29^. After 4 °C treatment for 6 days, 98.1% of the 2-week-old *ipa1-3D* seedlings survived; this rate was significantly higher than 65% for ZH11 seedlings (Fig. 4a, b). In contrast, the survival rate of *ipa1-10* seedlings dramatically dropped to 38.9% (Fig. 4a, b). The ion leakage analysis showed consistent results that *ipa1-3D* mutant had lower electrolyte leakage than ZH11 while *ipa1-10* had higher level under cold treatment (Fig. 4c). These results demonstrated that IPA1 was a positive regulator of chilling tolerance.

To explore the genetic interaction between OsSAPK6 and IPA1, we generated *ossapk6-3 ipa1-3D* double mutant by editing *OsSAPK6* in *ipa1-3D* background, which harbored the same mutation in *OsSAPK6* as *ossapk6-2* (Supplementary Fig. S1). *ossapk6-2* mutant was hypersensitive to chilling stress, while *ipa1-3D* mutant displayed increased chilling tolerance (Figs. 1a and 4a). The double mutant *ossapk6-3 ipa1-3D* exhibited similar phenotypes to *ipa1-3D*, including the high survival rate and low ion leakage under chilling stress (Fig. 4d–f). Meanwhile, we overexpressed *OsSAPK6* in the *ipa1-10* background and obtained *Ubi:OsSAPK6 ipa1-10* plants (Supplementary Fig. S5). *ipa1-10* plants was cold hypersensitive, and *Ubi:OsSAPK6* plants exhibited increased cold tolerance (Figs. 1d and 4a). Consistent with previous results, the *Ubi:OsSAPK6 ipa1-10* plants showed similar phenotypes to *ipa1-10* on chilling tolerance and ion leakage (Fig. 4g–i). These results indicate that OsSAPK6 and IPA1 work in same pathway in regulating chilling tolerance.

### Phosphorylation of IPA1 is critical for its function in chilling stress responses

**Fig. 5:**
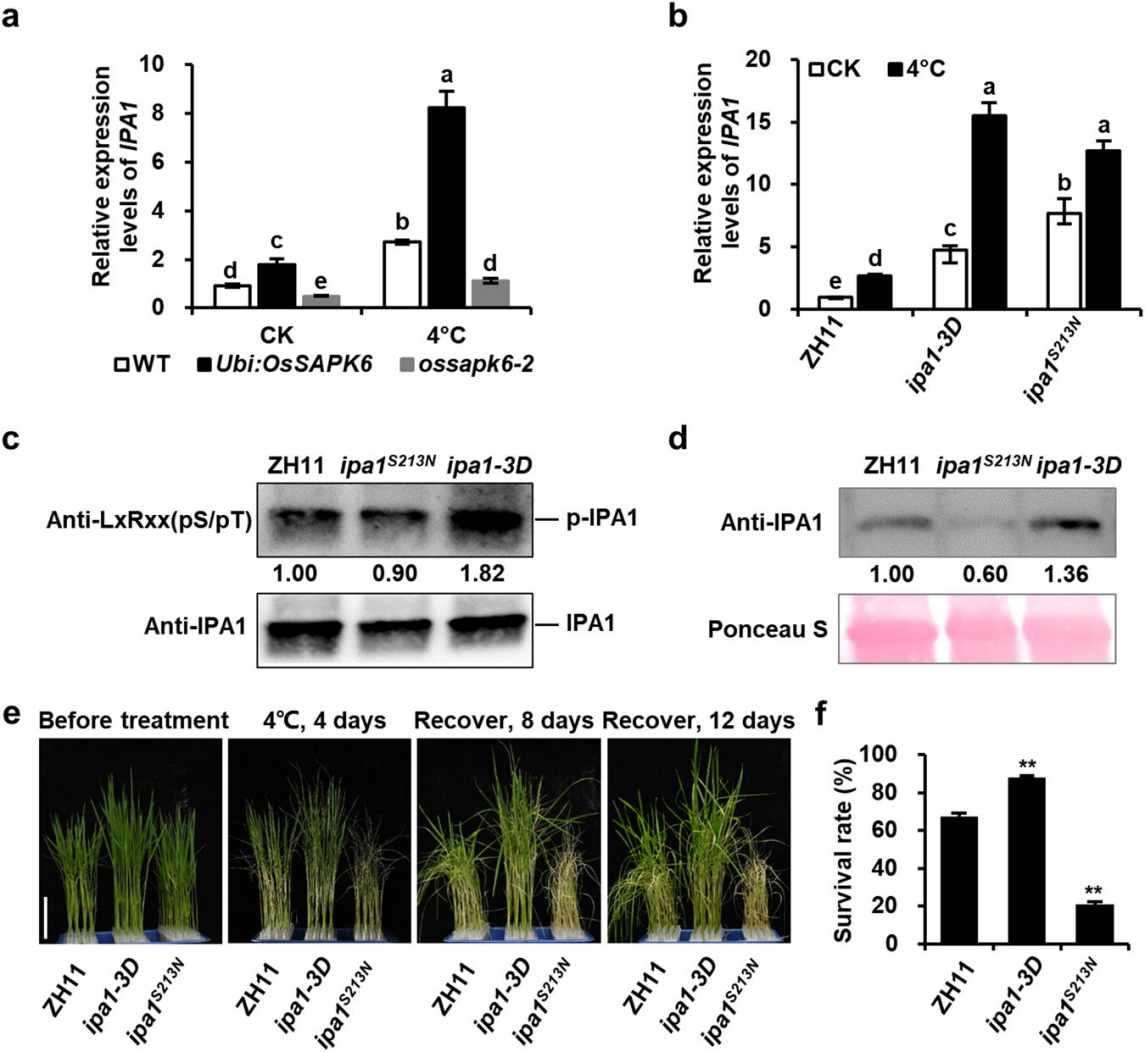
Phosphorylation of IPA1 is critical for its function in chilling stress responses. **a** Expression levels of *IPA1* in ZH11, *Ubi:OsSAPK6*, and *ossapk6-2* plants with or without 6 h 4 °C treatment. **b** *IPA1* expression levels in ZH11, *ipa1-3D* and *ipa1*^*S213N*^ after 4 °C treatment for 6 h. **c** The phosphorylation of IPA1 in ZH11, *ipa1-3D* and *ipa1*^*S213N*^. **d** IPA1 protein levels in ZH11, *ipa1-3D* and *IPA1*^*S213N*^. **e** Plant morphologies of 2-week-old ZH11, *ipa1-3D* and *ipa1*^*S213N*^ seedlings before treatment, after 4 °C treatment for 4 days and subsequent recovery for 8 or 12 days. Bars = 5 cm. **f** Survival rates of ZH11, *ipa1-3D* and *ipa1*^*S213N*^ after 4 °C treatment as shown in **e**. Values are means ± SD (*n* = 3 biological replicates). In **a** and **b**, the different letters indicate significant differences (*P* < 0.01) according to Tukey’s honest significant difference (HSD) test. In **f**, the asterisks indicate significant differences compared with ZH11 (**P < 0.01, Student’s *t*-test). See also Supplementary Fig. S6.

As *IPA1* expression level was increased in *OsSAPK6-OE*, we tested whether expression of *IPA1* could be induced by chilling stress. The Quantitative reverse transcription PCR (qRT-PCR) results showed that chilling-stress could induce the expression of *IPA1* in ZH11 as well as in *OsSAPK6-OE* and *ossapk6* (Fig. 5a), suggesting that OsSAPK6 may positively regulate the basal level of *IPA1* expression while *IPA1* expression under chilling stress was induced by other factors. As OsSAPK6 regulated *IPA1* at both transcriptional and post-transcriptional levels, we therefore tested whether the phosphorylation of IPA1 is critical for chilling tolerance. We used base editing strategy and designed a gRNA targeting the base pairs encoding Ser213 of IPA1, and obtained *ipa1*^*S213N*^ mutant line which harbored a C-to-T point mutation leading to the substitution of Ser213 to Asn (Supplementary Fig. S6). Compared to ZH11, the transcript level of *IPA1* was increased in *ipa1*^*S213N*^ mutant line while the phosphorylation level and protein level of IPA1 were both decreased (Fig. 5b–d). Moreover, under cold stress, *ipa1*^*S213N*^ line exhibited cold-sensitive phenotypes with lower survival rate as compared with WT (Fig. 5e, f). These results suggest that the phosphorylation of Ser213 is critical for IPA1 function in cold stress.

### IPA1 directly binds and activates *OsCBF3* promoter to regulate chilling stress responses

**Fig. 6:**
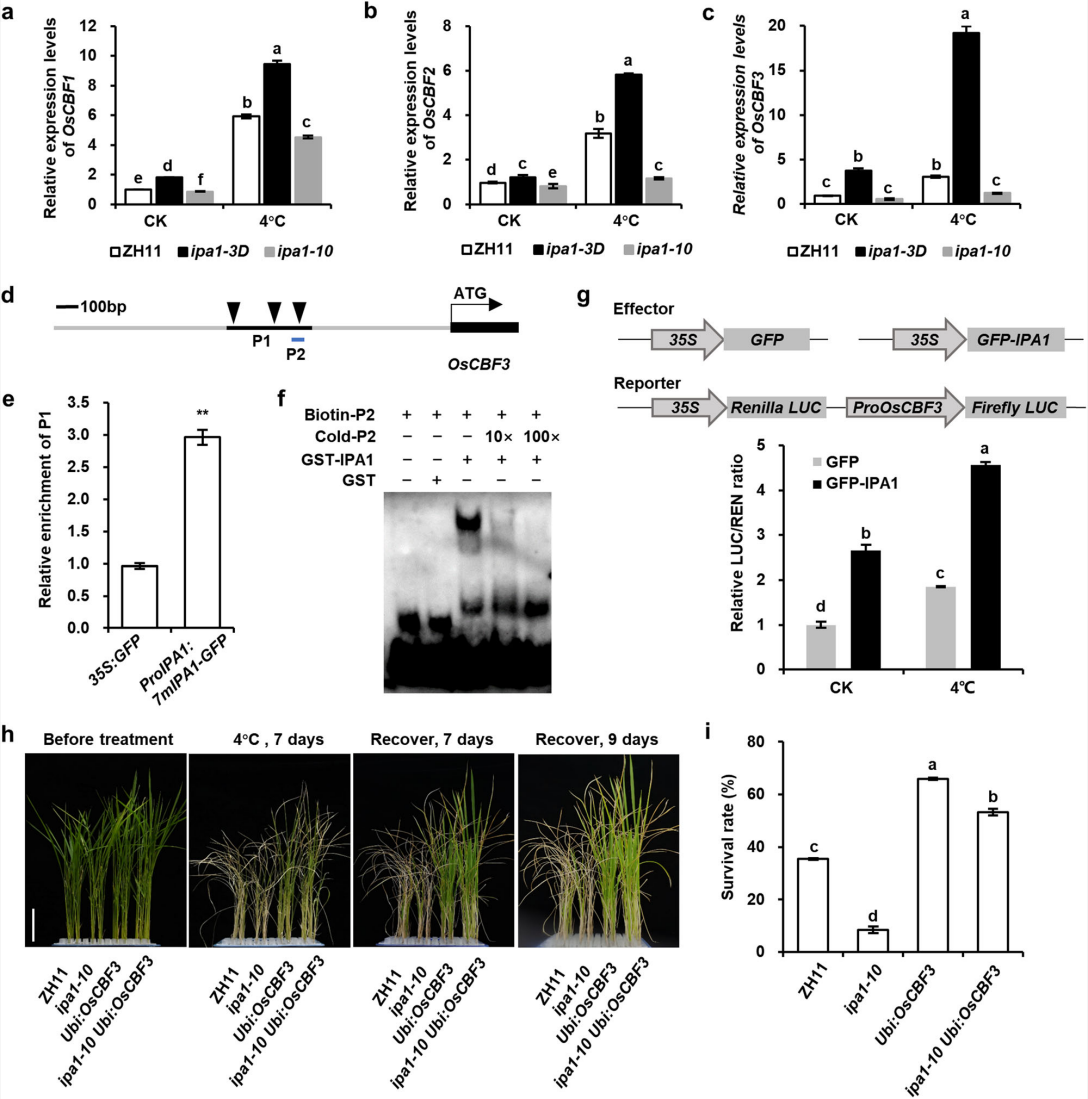
IPA1 directly binds to and activates *OsCBF3* promoter to regulate chilling stress response. **a**–**c** Expression levels of *OsCBF1* (**a**), *OsCBF2* (**b**), and *OsCBF3* (**c**) in ZH11, *ipa1-3D*, and *ipa1-10* plants under 25 °C (CK) or 4 °C for 6 h. **d** Schematics showing the promoter structure of *OsCBF3*. Solid arrowheads indicate GTAC motifs in the *OsCBF3* promoter. Hatched box P1 represents the fragment amplified in the ChIP-qPCR assay, and P2 represents the 42-bp fragment for EMSA. **e** ChIP-qPCR analysis of IPA1 binding sites (P1 in **d**) in the *OsCBF3* promoter with *ubiquitin* as a control. **f** Direct binding of IPA1 to the *OsCBF3* promoter in the EMSA assay. Biotin-labeled 42-bp fragment of the *OsCBF3* promoter (P2 in **d**) was incubated with GST or GST-IPA1 protein. **g** Transcriptional activity assay in rice protoplasts shows that IPA1 could activate the expression of *OsCBF3*. *ProOsCBF3:LUC* was co-transformed with *GFP* or *GFP-IPA1* effector for 12 h and then incubated at 28 °C (CK) or 4 °C for 1 h. **h** Plant morphologies of 2-week-old ZH11, *ipa1-10*, *Ubi:OsCBF3*, and *ipa1-10 Ubi:OsCBF3* seedlings before treatment, after 4 °C treatment for 7 days, and subsequent recovery for 7 or 9 days. **i** Survival rates of ZH11, *ipa1-10*, *Ubi:OsCBF3*, and *ipa1-10 Ubi:OsCBF3* after 4 °C treatment as shown in **h**. In **a**–**c**, **g** and **i**, values are means ± SD (*n* = 3 biological replicates). Different letters indicate significant differences (*P* < 0.01) according to Tukey’s HSD test. In **e**, values are means ± SD (*n* = 3 technical replicates). At least three independent experiments were performed with similar results, the asterisks indicate significant differences compared with the control (**P < 0.01, Student’s *t*-test). Bars = 5 cm in **h**. See also Supplementary Figs. S7–S10 and Table S2.

To identify potential target genes of IPA1 under chilling stress, we performed RNA-seq using 2-week-old WT and *ipa1-3D* seedlings under normal growth condition or 6 h 4 °C treatment (Supplementary Fig. S7a and Table S2). The result showed that most of the cold-induced genes in WT was also highly induced in *ipa1-3D*, while the cold-repressed genes were less overlapped (Supplementary Fig. S7b, c). Moreover, among the highly induced genes (adjusted *P* value < 1e-10), we found 13 genes whose expression levels were further enhanced under chilling treatment in *ipa1-3D* compared to WT, including *OsCBF2* and *OsCBF3* in the top five list (Supplementary Fig. S7d). In *Arabidopsis*, cold-induced expression of *CBFs* played a central role in cold stress^1,7^. In rice, *OsCBF3* could positively regulate cold tolerance^13^. We therefore examined the expression levels of *OsCBFs*/*OsDREB1s* in *IPA1* mutants in detail. Under the normal condition, the expression levels of *OsCBF1* and *OsCBF3* were significantly higher in the gain-of-function mutant *ipa1-3D* and lower in loss-of-function mutant *ipa1-10* than those in ZH11 (Fig. 6a–c; Supplementary Fig. S8). After chilling treatment, the expressions of *OsCBF1*, *OsCBF2*, and *OsCBF3* were all induced in ZH11, and the induction of *OsCBF3* expression was strongly enhanced in *ipa1-3D* but alleviated in *ipa1-10*. These results indicate that *OsCBF* may function in the downstream of IPA1 in regulating chilling tolerance.

As *OsCBF3* showed the strongest chilling-induced expression in *ipa1-3D*, we therefore examined whether IPA1 could directly regulate *OsCBF3* expression. Since IPA1 could directly bind to GTAC motif^30^, we focused on GTAC motif and found three GTAC sequences 2-kb upstream of *OsCBF3* (Fig. 6d). By using the primers targeting the GTAC-containing region P1 in the promoter of *OsCBF3*, we conducted a ChIP-qPCR assay and found that this region could truly be enriched in *ProIPA1:7mIPA1-GFP* transgenic plants but not in *35* *S:GFP* transgenic plants (Fig. 6e). To test whether IPA1 could directly bind to the promoter of *OsCBF3*, we conducted an electrophoretic mobility shift assay (EMSA) using the recombinant IPA1-GST protein expressed in and purified from *E. coli*. The result showed that the GST-IPA1 protein was able to bind to the 42-bp GTAC-containing region P2 in the *OsCBF3* promoter, but no signal was observed for the control GST protein (Fig. 6f). In addition, the signal intensity of retarded bands decreased in the presence of increasing concentrations of unlabeled competitor probe, whereas no binding was detected when adding the mutated DNA probes containing ATAC instead of GTAC (Fig. 6f; Supplementary Fig. S9). Moreover, we found that IPA1 could activate the reporter gene expression driven by the promoter of *OsCBF3* using the transcriptional activity assay in rice protoplasts, and the expression level of the reporter gene was further induced under chilling stress (Fig. 6g). Under chilling stress, OsCBF3 could directly bind to and activate the *OsCNGC9* promoter^13^. Consistent with this, we found *OsCNGC9* expression was upregulated in *ipa1-3D* and down-regulated in *ipa1-10* (Supplementary Fig. S10). All these data indicate that IPA1 can directly bind to and activate the promoter of *OsCBF3*.

To further explore the genetic interaction between *IPA1* and *OsCBF3*, we generated *Ubi:OsCBF3* and *ipa1-10 Ubi:OsCBF3* lines by introducing *Ubi:OsCBF3* into ZH11 and *ipa1-10* plants. *Ubi:OsCBF3* was chilling tolerant as previously reported^13^, and *ipa1-10* was chilling sensitive (Figs. 4a and 6h). The 2-week-old *ipa1-10 Ubi:OsCBF3* plants showed chilling tolerant phenotypes similar to *Ubi:OsCBF3*, and the survival rate was higher than ZH11 but still lower than *Ubi:OsCBF3* after cold treatment (Fig. 6h, i). Taken together, all these data suggest that *OsCBF3* is directly activated by IPA1 in chilling tolerance pathway.

### OsSAPK6-IPA1-OsCBF signaling cascade under chilling stress

**Fig. 7:**
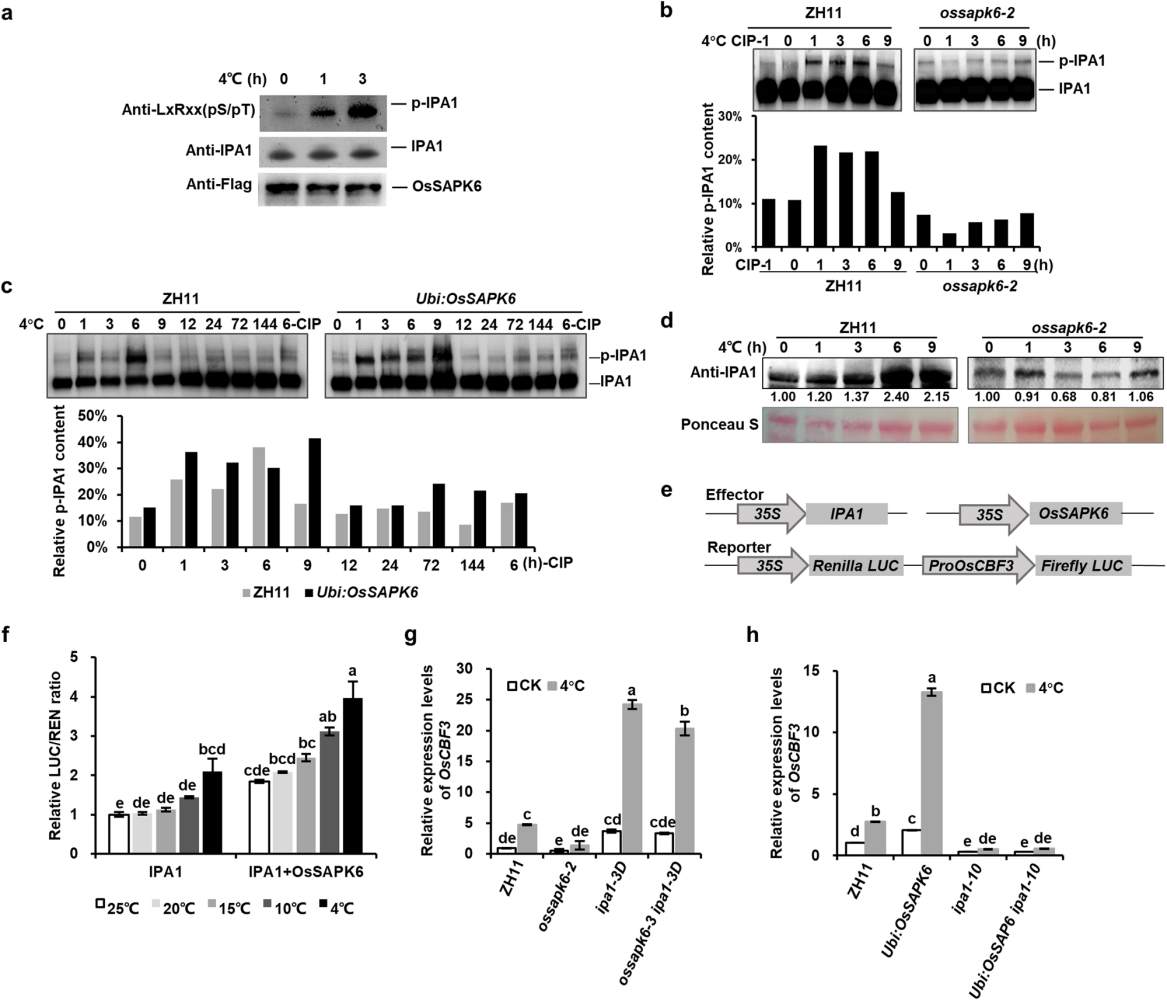
OsSAPK6 phosphorylates IPA1 under chilling stress to upregulate *OsCBF3* expression. **a** OsSAPK6 kinase activities under chilling stress. *Flag-OsSAPK6*-*OE* transgenic seedlings were treated under 4 °C for the indicated time, and Flag-OsSAPK6 protein was then purified with anti-Flag beads, normalized, and incubated with equal amounts of purified GST-IPA1 for 30 min. The phosphorylated GST-IPA1 was detected with a polyclonal antibody against a substrate motif [LXRXX (pS/pT)], and GST-IPA1 was detected with anti-IPA1 monoclonal antibody. **b** Relative content of phosphorylated IPA1 in ZH11 and *ossapk6-2* seedlings under chilling stress. Protein extracts from ZH11 and *ossapk6-2* seedlings were separated in a Phos-tag gel, and the phosphorylated and nonphosphorylated IPA1 proteins were detected with anti-IPA1 antibody and quantitated by densitometry. **c** Relative content of phosphorylated IPA1 in ZH11 and *OsSAPK6*-OE seedlings under chilling stress. **d** IPA1 protein levels in ZH11 and *ossapk6-2* seedlings under chilling stress. IPA1 protein was detected by anti-IPA1 antibody. Relative amounts of proteins were determined by densitometry and normalized to loadings determined by Ponceau staining (red). **e** Schematics of constructs used for transcriptional activity assays in rice protoplasts. **f** Transcriptional activity assay in rice protoplasts showing that IPA1-mediated activation of *OsCBF3* expression was promoted by OsSAPK6 and temperature sensitive. **g** Expression levels of *OsCBF3* in ZH11, *ossapk6-2*, *ipa1-3D*, and *ossapk6-3 ipa1-3D* after 4 °C treatment for 6 h. **h** Expression levels of *OsCBF3* gene in ZH11, *Ubi:OsSAPK6*, *ipa1-10*, and *Ubi:OsSAPK6 ipa1-10* after 4 °C treatment for 6 h. In **f**–**h**, values are means ± SD (*n* = 3 technical replicates). At least three independent experiments were performed with similar results. Different letters indicate significant differences (*P* < 0.01) according to Tukey’s HSD test. See also Supplementary Figs. S11–13.

To investigate the signal transduction of chilling stress responses, we investigated the function of cold-induced phosphorylation of IPA1 by OsSAPK6 in detail. As abiotic and biotic stresses could activate protein kinase^18,31–33^, we first examined whether OsSAPK6 autophosphorylation and kinase activity was affected by chilling stress. Indeed, cold stress could induce the autophosphorylation of OsSAPK6 (Supplementary Fig. S11a). Then 2-week-old *Flag-OsSAPK6-OE* transgenic plants were chilling treated for 1 or 3 h(s), and OsSAPK6 proteins were purified from these plants using anti-Flag beads, which were then incubated with IPA1 for testing the phosphorylation levels of IPA1. Indeed, we found that the kinase activity of OsSAPK6 was increased in plants with 1 h chilling treatment and further increased in plants with 3 h treatment (Fig. 7a; Supplementary Fig. S11b, c). These data suggest that chilling stress can induce the kinase activity of OsSAPK6.

We then examined whether IPA1 was phosphorylated and stabilized by OsSAPK6 under cold stress. After cold treatment, the phosphorylation level of IPA1 was significantly increased in ZH11, but this phenomenon was dramatically suppressed in the *ossapk6-2* mutant (Fig. 7b). To examine the dynamic phosphorylation levels of IPA1, we treated WT and *SAPK6-OE* plants with cold stress for 6 days, and found that in WT the relative content of phosphorylated IPA1 was elevated within 6 h and then went down, while in *SAPK6-OE* plants phosphorylated IPA1 was elevated until 9 h with a higher extent and went down afterward (Fig. 7c), suggesting that *SAPK6-OE* could trigger higher level of IPA1 phosphorylation with longer duration. Moreover, the phosphorylated IPA1 was detected as early as two minutes after chilling treatment, indicating this was a quick response to chilling stress (Supplementary Fig. S11d). The phosphorylation of IPA1 was abolished with the addition of calf-intestinal alkaline phosphatase (CIP) (Fig. 7b, c; Supplementary Fig. S11d). We then examined the protein levels of IPA1 in ZH11 and *ossapk6-2* plants after chilling stress, and found that the protein level of IPA1 was gradually accumulated under chilling treatment in ZH11 but not in *ossapk6-2* (Fig. 7d; Supplementary Fig. S11e). Taken together, these results indicate that OsSAPK6 can phosphorylate and stabilize IPA1 under cold stress.

We further tested whether OsSAPK6 can affect the transcriptional activation of *OsCBF3* by IPA1, and found that co-expression of *OsSAPK6* with *IPA1* could increase the expression of reporter genes driven by the promoter of *OsCBF3* in transcriptional activity assay in rice protoplasts (Fig. 7e, f). Notably, this effect exhibited a temperature sensitive pattern that the induction of reporter gene expression by OsSAPK6 and IPA1 was positively associated with the decrease of the temperature (Fig. 7f). We then examined the expression levels of *OsCBF3* in *OsSAPK6*- and *IPA1*- related mutants. Compared with ZH11 in normal condition, the expression levels of *OsCBF3* were decreased in *ossapk6-2* and *ipa1-10*, but increased in *Ubi:OsSAPK6* and *ipa1-3D* (Fig. 7g, h). Under chilling stress, the expression of *OsCBF3* was induced in ZH11, and this induction was declined in *ossapk6-2* and *ipa1-10*, and strongly enhanced in *Ubi:OsSAPK6* and *ipa1-3D* (Fig. 7g, h). The double mutant *ossapk6-3 ipa1-3D* showed a similar pattern of *OsCBF3* expression to *ipa1-3D* plant under both normal and chilling stress conditions, while *Ubi:OsSAPK6 ipa1-10* double mutant showed similar pattern to *ipa1-10*, indicating that the positive regulation of *OsCBF3* expression by OsSAPK6 is dependent on IPA1 under cold stress (Fig. 7g, h). The expression levels of three randomly selected genes were also tested as negative controls (Supplementary Fig. S12).

In addition, we treated the 2-week-old seedlings of WT, *Ubi:OsSAPK6*, *ipa1-10*, and *Ubi:OsSAPK6 ipa1-10* with heat stress of 35 °C, and found that the cold-resistant *Ubi:OsSAPK6* also showed heat-resistant phenotype while cold-sensitive mutant *ipa1-10* was heat-sensitive, and that *Ubi:OsSAPK6 ipa1-10* showed heat-sensitive phenotype similar to *ipa1-10* (Supplementary Fig. S13a, b). Consistent with this, *ossapk6-2* exhibited heat-sensitive phenotype and *ipa1-3D* showed heat-resistant phenotype, while *ossapk6-3 ipa1-3D* showed a higher resistant than *ossapk6-2* (Supplementary Fig. S13c, d). However, the expression level of *OsCBF3* was not induced under heat stress but significantly repressed (Supplementary Fig. S13e), suggesting that *OsSAPK6* and *IPA1* but not *OsCBF3* may also function in heat stress tolerance. Taken together, all these data indicate that OsSAPK6-IPA1-OsCBF functions as a chilling-induced gene cascade to regulate chilling tolerance in rice.

### Natural allele *ipa1-2D* increases grain yield and seedling chilling tolerance

**Fig. 8:**
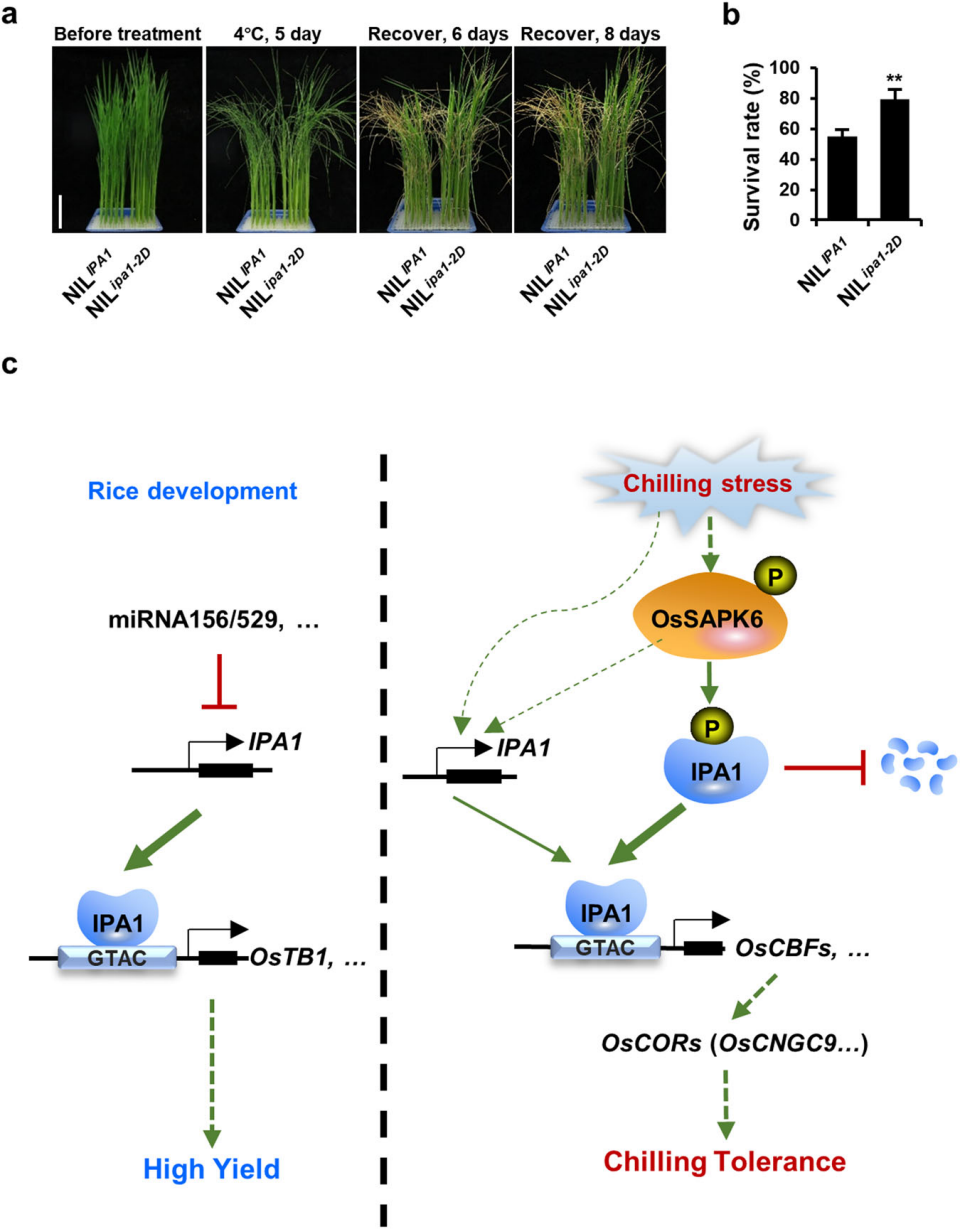
Natural allele *ipa1-2D* enhances seedling chilling tolerance. **a** Plant morphologies of 2-week-old NIL^*IPA1*^ and NIL^*ipa1-2D*^ seedlings before treatment, after 4 °C treatment for 5 days and subsequent recovery for 6 or 8 days. **b** Survival rates of NIL^*IPA1*^ and NIL^*ipa1-2D*^ after 4 °C treatment as shown in **a**. Values are means ± SD (*n* = 3 biological replicates), and the asterisks indicate significant differences compared with the NIL^*IPA1*^ (**P < 0.01, Student’s *t-*test). **c** Proposed model of OsSAPK6-IPA1-OsCBFs signaling cascade under chilling stress in rice. Under chilling stress, OsSAPK6 phosphorylates IPA1 on Ser201 and Ser213, leading to an accumulation of IPA1 proteins, and IPA1 in turn up-regulates *OsCBFs* expression by binding to GTAC motifs on the *OsCBFs* promoter, which then activates the downstream *COR* genes such as *OsCNGC9* to confer chilling tolerance in rice.

*IPA1* was regarded as a new “green revolution” gene for its significant potential in enhancing grain yield^34^, and its natural elite gain-of-function alleles, especially the *ipa1-2D*, have made great contributions in breeding new super hybrid rice varieties^22,35,36^. The *ipa1-2D* contains a 3,137-bp tandem repeat in the upstream of *IPA1*, which can elevate the expression of *IPA1* and increase the stem thickness, grain number, and yield. We therefore tested whether this allele can increase seedling chilling tolerance with the previously generated high-quality near-isogenic lines (NILs) NIL^*IPA1*^ and NIL^*ipa1-2D*^ in the Nipponbare (NIP) background^35^. After chilling stress and recovery, the survival rate of 2-week-old NIL^*ipa1-2D*^ seedlings was significantly higher than that of NIL^*IPA1*^ (Fig. 8a, b), indicating that the rice varieties with *ipa1-2D* can benefit from both increased grain production and enhanced chilling tolerance in seeding stage.

## Discussion

Although the cold signal transduction has been well established in *Arabidopsis*^1,7,8^, the understanding of chilling stress response in rice remains largely unknown. In this study, we revealed a chilling-induced OsSAPK6-IPA1-OsCBF cascade in triggering rice chilling tolerance. First, OsSAPK6, a member of SnRK2 family, could positively regulate cold responses in rice, whereas the overexpression of *OsSAPK6* increased the chilling tolerance of rice and its loss-of-function mutant displayed a chilling-sensitive phenotype (Figs. 1 and 4). Second, under chilling stress, OsSAPK6-mediated phosphorylation of IPA1 stabilized IPA1 and resulted in enhanced *OsCBF* expression and rice chilling tolerance (Figs. 2, 3, and 5–7). Third, IPA1 could directly bind to and activate the transcription of *OsCBF3*. The genetic evidence illustrated that OsSAPK6, IPA1 and OsCBF3 worked in the same pathway in rice chilling responses (Figs. 6 and 7).

In *Arabidopsis*, ICE1 was the key transcription factor in *CBF*-dependent transcriptional regulatory pathway in cold responses^9–11,37^. ICE1 directly upregulated *CBF* expression and freezing tolerance^9,33^. Cold-activated protein kinase OST1 phosphorylated ICE1 to inhibit its degradation mediated by the E3 ligase HOS1, which in turn enhanced ICE1 stability and transcriptional activity^11^. Here, we found that IPA1 was the key transcription factor in the rice *CBF*-dependent pathway to regulate chilling responses (Figs. 4 and 6). IPA1 was phosphorylated and stabilized by the chilling-activated protein kinase OsSAPK6 (Figs. 2 and 7), and directly upregulated *OsCBF3* expression (Fig. 7h, i). OsSAPK6 and OST1 belong to different subfamilies of the SnRK2 protein family, and the closest homolog of OST1, OsSAPK8, also positively regulates chilling tolerance in rice by phosphorylating OsCNGC9^13,27^. ICE1 is a MYC-like bHLH transcriptional activator binding to MYC recognition sequences in the promoter of *CBFs* in *Arabidopsis*, while IPA1 is a member of the SPL family targeting GTAC motif in the promoter of *OsCBF3* in rice^9–11,30^. The regulatory mechanism of cold-induced signal cascade mediated by protein kinase, transcription factors, and responsive genes might be conserved in both monocotyledonous and dicotyledonous plants. ICE1 is degraded by the E3 ligase HOS1, which could be inhibited by OST1-mediated phosphorylation under cold stress^11^. IPA1 Interaction Protein 1 (IPI1), a RING-finger E3 ligase, was previously reported to promote the degradation of IPA1 in panicles and stabilize IPA1 in shoot apexes to regulate plant architecture^38^. Whether IPI1-mediated degradation of IPA1 is inhibited in the chilling-induced phosphorylation of IPA1 by OsSAPK6 remains to be elusive.

IPA1 is a pleiotropic gene playing critical roles in multiple aspects of rice development and stress responses^22–24^. The stress-induced modification of IPA1 protein played important roles in stress responses^23,24^. For rice blast response, IPA1 was phosphorylated on Ser163 and in turn upregulated *WRKY45* to enhance rice blast resistance^23^, whereas the responsible kinase was still unknown. In this study, we found that IPA1 was phosphorylated on Ser201 and Ser213 by OsSAPK6 to upregulate *OsCBF3* to enhance rice chilling tolerance, and the phosphorylation of IPA1 was critical for its function in chilling stress responses (Figs. 2h, 5, and 7g). The precise regulation of IPA1 mediated by phosphorylation modification renders the diversity of IPA1 protein functions. As generally considered, plants have evolved a trade-off regulatory mechanism to balance the growth and resistance to stresses. One possible mechanism is that plant can induce the transient modification of key multi-function proteins under stress. For example, IPA1 could be transiently phosphorylated under abiotic and biotic stresses, however, the downstream common or specific signaling pathway remains elusive. The further illustration of the phosphorylation sites on IPA1, the upstream kinase and the effects on downstream target genes will provide valuable knowledge in understanding the relationship between rice development and stress responses.

Ca^2+^ channel is another key component in cold signal transduction^13,39^. Cold stress could alter the fluidity of cellular membranes and induce a rapid and transient increase in Ca^2+^ influx in rice^13,14,40^. Ca^2+^ influx was proposed as an early event in the cold-stress response which happened in seconds after cold stress^40,41^. Cold signal could be sensed by the membrane protein complexe COLD1–RGA1 to induce Ca^2+^ influx and activate *OsCBFs* expression^14,33^. However, the exact molecular function of COLD1 in regulating Ca^2+^ influx and regulatory relationships between Ca^2+^ influx and *OsCBFs* are still unknown. Recently, OsCNGC9, a Ca^2+^-permeable non-specific cation channel, was reported to positively regulate rice chilling tolerance by mediating Ca^2+^ influx^13,14^. Under chilling stress, the OsCNGC9 protein was phosphorylated by OsSAPK8 in minutes, and the expression of *OsCNGC9* could also be directly activated by OsCBF3/DREB1A^13^. Therefore, the Ca^2+^ channel OsCNGC9 acted downstream of OsSAPK8 and OsCBF3 in response to chilling stress at post-translational level and transcriptional level^13^. In this study, we found that IPA1 could be phosphorylated in minutes after chilling stress, leading to the accumulation of IPA1 protein and up-regulation of *OsCBF3* and *OsCNGC9* expression in hours (Figs. 2 and 6). Transient Ca^2+^ influx, activation of protein kinase, and transcriptional regulation were observed to happen respectively in seconds, minutes and hours after chilling stress, which are all critical for chilling tolerance. Complex feedback loops seem to form in chilling responses that the chilling-activated protein kinases and transcription factors can also regulate the Ca^2+^ channel. The relationship between Ca^2+^ influx and protein kinase activity under chilling stress remains to be elucidated.

Based on these data, we proposed a model that OsSAPK6 acts in the upstream of IPA1 in response to chilling stress to positively regulate rice chilling tolerance through the *OsCBF*-dependent pathway (Fig. 8c). Our results have revealed a comprehensive *CBF*-dependent transcriptional regulatory pathway in chilling stress responses in rice. Moreover, the natural gain-of-function allele *ipa1-2D* promoted both rice yield and chilling tolerance, which provided valuable genetic resources for rice breeding.

## Materials and methods

### Plant materials and growth conditions

Rice (*Oryza sativa* L. subsp. *japonica*) ZH11, *ipa1-3D*, *ipa1-10*, *ossapk6-1/2*, *ossapk6-3 ipa1-3D* double mutant, as well as *OsSAPK6* overexpression (*Ubi:OsSAPK6*) lines were grown either in the controlled growth chamber (MLR-352H-PC, Panasonic) under 16-h day/8-h night at 25 °C/16 °C (day/night) cycles or in the experimental field of the Institute of Genetics and Developmental Biology in Beijing. Double mutant of *ossapk6-3 ipa1-3D* was generated by CRISPR/Cas9 using the vector VK005 (VK005-01, Beijing Viewsolid Biotech) from the *ipa1-3D* background. The *ipa1*^*S213N*^ mutant line was generated by CRISPR/Cas9 using the vector pZRH-PBE from the ZH11 background. For *OsSAPK6* overexpression lines, full-length cDNA of *OsSAPK6* was cloned into pJL1460 vector and driven by *Ubiquitin* promoter to generate the *Ubi:OsSAPK6* construct. *Agrobacterium tumefaciens* (strain EHA105)-mediated transformation was used to introduce the constructs into rice^42^. Positive lines were confirmed by PCR followed by sequencing, and then the homozygous T_2_ plants were used for the experiments. All primers and gRNAs used in the present study are listed in Supplementary Table S3.

### Chilling and heat tolerance assays

Rice seeds were soaked in water for 3 days at 37 °C. The germinated seeds were then placed into an incubator with Kimura B nutrient solution and grown in a plant growth chamber under 16-h day/8-h night at 25 °C/16 °C (day/night) cycles. Two-week-old seedlings were used for the subsequent tolerance assays. Chilling tolerance assays were performed as described^13^ with modifications. The seedlings were moved into a plant growth chamber (MLR-352H-PC, Panasonic) maintained at 4 ± 0.5 °C for a chilling treatment. For heat treatment, the seedlings were moved into the plant growth chamber (MLR-352H-PC, Panasonic) maintained at 35 ± 0.5 °C for a heat treatment. The duration of the treatment varied from four to seven days. Subsequently, seedlings were returned to plant growth chamber conditions and allowed to recover for four to fifteen days. The survival rate (percentage of live seedlings) was determined. Each experiment was conducted independently at least three times.

### Ion leakage assay

The chilling-treated seedlings were harvested for ion leakage assays^11^. Leave samples from 5 plants (0.3 g) were immersed in a 15-mL tube containing 5 mL deionized water and shaken at 200 rpm at room temperature for 1 h, and the electrical conductivity (S1) was determined. The samples were boiled at 100 °C for 20 min, shaken at 22 °C for 1 h, and then detected to determine the total conductivity (S2). The electrical conductivity of deionized water was defined as S0. Relative ion leakage was calculated as (S1‒S0)/(S2‒S0).

### qRT-PCR

RNA isolation, reverse transcription and real-time PCR were performed as described previously^29,43^. Total RNAs were extracted from rice tissues using TRIzol reagent (15596018, Life), followed by reverse transcription using a QuantiTect Reverse Transcription Kit (205313, Qiagen). qRT-PCR was performed using SYBR Green Kit (208054, Qiagen) in a real-time PCR system (CFX96, Bio-Rad). Three biological replicates were set up, and each sample was analyzed at least in triplicate. Primers used are listed in Supplementary Table S3.

### Subcellular localization assays

To determine the subcellular localization of IPA1, IPA1^201A213A^, and OsSAPK6, the full-length coding sequences of the corresponding genes were amplified and fused to GFP in the plant expression vector pBI221 (HonorGene). Rice leaf protoplast isolation and transformation were carried out according to the protocol as previously described^44^. *IPA1*, *IPA1*^*201A213A*^, and *OsSAPK6* together with *35* *S:mCherry* (marker for nucleus) were co-transformed into rice leaf protoplasts. *35S:GFP* was used as a control. After incubation in the dark overnight, the localization patterns were assessed by visualizing GFP fluorescence using a confocal laser-scanning microscope (Zeiss LSM-710). The primers used are listed in Supplementary Table S3.

### BiFC assays

The coding sequence of *IPA1* or *IPA1*^*201A213A*^ was cloned into puc-SCYCE, and *OsSAPK6* was cloned into puc-SCYNE. The plasmid mixtures were introduced into rice leaf protoplasts as described^44^. After incubation in the dark overnight, the fluorescence was observed with a confocal laser-scanning microscope (Zeiss LSM-710).

### Yeast two-hybrid assays

Yeast two-hybrid assays were performed using the Matchmaker GAL4-based Two-Hybrid System 3 (Clontech) following the manufacturer’s instructions. Constructs were produced by cloning *OsSAPK6* into the vector pGADT7 (Takara Bio Inc.) and *IPA1* into pGBKT7 (Takara Bio Inc.). The truncation versions for IPA1 amino acids 1–103, 1–181, 104–417, and 182–417, were each inserted into pGBKT7. All different combinations of the prey and bait constructs were co-transformed into yeast strain AH109 by the lithium acetate method^29,45^. After transfection, strains were cultured on minimal medium/-Leu/-Trp, further selected on minimal medium/-Leu/-Trp/-His/-Ade. The primers used for the yeast two-hybrid assays are provided in Supplementary Table S3.

### Co-IP assay

The total proteins were extracted from rice protoplasts expressing *35S:HA-IPA1/35S:GFP* or *35S:HA-IPA1*/*35S:OsSAPK6-GFP* constructs and immunoprecipitated with anti-IPA1 antibody. Proteins were detected with anti-GFP (ab290, Abcam) antibody.

### Antibody production

To detect the IPA1 protein in rice, we obtained IPA1-specific antibody against a synthetic peptide (amino acids 1–96). The IPA1 monoclonal antibody was produced by HUABIO Co., Ltd (Hangzhou, Zhejiang, China). The specificity of anti-IPA1 antibody was validated using wild-type and *IPA1-GFP* transgenic lines (Supplementary Fig. S7a).

### Phos-tag mobility shift assay

Phos-tag reagent (AAL-107, Wako) was used for the phosphoprotein mobility-shift assay to detect phosphorylated IPA1 proteins as described^46^. For the in vitro phosphorylation assay, purified GST-IPA1 and His-OsSAPK6 were incubated in kinase buffer containing 20 mM Tris-HCl (pH 7.5), 10 mM MgCl_2_, and 10 mM ATP at 30 °C for 30 min. The samples were then incubated with or without CIP at 37 °C for 30 min, and the proteins were separated in a 12% (w/v) SDS-PAGE gel containing 50 μM Phos-tag and 100 μM MnCl_2_. The gel was incubated in transfer buffer containing 10 mM EDTA three times and then washed in transfer buffer for 10 min. After transferring onto PVDF membranes, the GST-IPA1 protein was detected with an anti-GST (M20007L, Abmart) monoclonal antibody.

For the in vivo phosphorylation assay, ZH11, *ossapk6-2*, or *IPA1-GFP* transgenic plants were treated with chilling stress and proteins were extracted with extraction buffer. The samples were incubated with or without CIP at 37 °C for 30 min, and then analyzed using 12% (w/v) SDS-PAGE gel containing 50 μM Phos-tag and 100 μM MnCl_2._ After transferring into PVDF membranes, the IPA1 protein was detected with an anti-IPA1 monoclonal antibody.

### Cell-free protein degradation assay

Cell-free protein degradation assay was performed as described^11^ with some modifications. Total proteins were extracted from 2-week-old seedlings of wild-type ZH11, *ossapk6-2*, and *Ubi:OsSAPK6* plants with extraction buffer. Equal amounts of above total proteins were incubated with equal amounts of recombinant GST-IPA1, GST-IPA1^S201A^, GST-IPA1^S213A^, or GST-IPA1^S201AS213A^ protein and 10 mM ATP for the indicated time. The proteins were separated by SDS-PAGE and detected with anti-GST monoclonal antibody. Rice rubisco large subunit or actin was used as a loading control.

### Protein extraction and immunoblot analysis

For protein extraction, rice tissues were ground in liquid nitrogen and proteins were extracted using the extraction buffer containing 100 mM Tris-HCl, pH 7.5, 100 mM KCl, 10% glycerol, 2 mM DTT, 1 mM phenylmethylsulfonyl fluoride (PMSF), 1% Triton X-100, and 1× protease inhibitor cocktail. The total extraction was mixed thoroughly and centrifuged at 12,000 × *g* and 4 °C for 20 min. The suspension was transferred to a new tube. The concentration of proteins was measured using a BCA Protein Assay Kit (23227, Pierce) and finally the protein concentration of each sample was adjusted to the same level. For immunoblotting, proteins were separated by 10% (w/v) SDS-PAGE and transferred to supported nitrocellulose transfer membrane (1704150, Bio-Rad) by electro-transfer at 70 V for 30 min. The membrane was blocked in TBST buffer containing 5% skim milk powder and further incubated with primary antibody for 120 min at room temperature and then secondary antibody for 60 min. Finally, bands on the blot were detected using chemiluminescent HRP substrate (RPN2235, Cytiva). Relative amounts of proteins were determined by densitometry and normalized to loadings using Image J software.

The antibodies used in this study included anti-GFP (ab290, Abcam), anti-GST (M20007L, Abmart), anti-His (M30111, Abmart), anti-Actin (M20009L, Abmart), and anti-LXRXX(pS/pT) (5759, CST).

### Transcriptional activity assay in rice protoplasts

The plasmid pGreen0800-*ProOsCBF3*-*LUC* combined with *35S: IPA1-GFP*, *35S: IPA1-GFP*, or *35S: OsSAPK6-Flag* were introduced into rice leaf protoplasts as described^42^. After incubation in the dark overnight, the luciferase activities were measured by the Dual-Luciferase Reporter Assay System (E1910, Promega) according to the manufacturer’s instructions.

### ChIP-qPCR assay

Two-week-old *ProIPA1:7mIPA1-GFP* transgenic seedlings were used for ChIP-qPCR assays, which were performed as previously described^47^. The enriched DNA fragments were analysed by qRT-PCR using the primers as listed in Supplementary Table S3. PCR reactions were performed in triplicate for each sample, and the expression levels were normalized to the *ubiquitin* (LOC_Os03g13170) promoter. No addition of antibodies (NoAbs) was served as a negative control.

### EMSA assay

Probe labeling and EMSA were performed as described previously^29^. The amplified CDS of *IPA1* was fused in frame with the GST tags in the pGEX-4t-1 vector (Crgen). The GST-IPA1 recombinant protein was expressed in *E. coli* BL21 (DE3) cells at 16 °C for 10 h using 0.3 mM IPTG and purified with Glutathione Sepharose 4B (17-0756-01, GE Healthcare). Oligonucleotide probes of *OsOsCBF3* were synthesized and labeled with 5′-biotin (Invitrogen) by annealing of complementary oligonucleotides at 72 °C for 30 min. EMSA was performed using a LightShift® Chemiluminescent EMSA Kit (20148, Thermo Scientific) according to the manufacturer’s instructions. Probes used are listed in Supplementary Table S3.

### RNA-seq analysis

The paired-end clean reads of RNA-seq were aligned to the rice reference genome Os-Nipponbare-Reference-IRGSP-1.0 using STAR (version 2.4.2a)^48,49^. Fragment quantifications were computed with FeatureCounts (version 1.5.0) in paired-end mode, the features are exons^50^. Expression differentiation analyses were conducted using the R (version 3.3.1) package DESeq (version 1.26.0) with three biological replicates^51^.

### Accession numbers

Gene sequences used in this study can be found in the Rice Genome Annotation Project under following accession numbers: *IPA1* (LOC_Os08g39890), *OsSAPK6* (LOC_Os02g34600), *OsSAPK8* (LOC_Os03g55600), *OsCBF1* (LOC_Os09g35010), *OsCBF2* (LOC_Os06g03670), *OsCBF3* (LOC_Os09g35030), and *OsCNGC9* (LOC_Os09g38580).

## Supplementary information

Supplementary Figures

Supplementary Table 1

Supplementary Table 2

Supplementary Table 3

## Data Availability

Raw sequencing data of RNA-seq have been deposited into the Genome Sequence Archive (GSA) database in BIG Data Center under Accession Number CRA006382.
