# Supplementary material for: Chilling-induced phosphorylation of IPA1 by OsSAPK6 activates chilling tolerance responses in rice
Source: Cell Discov. 2022 Jul 26;8:71. doi: 10.1038/s41421-022-00413-2 (PMC9325753; doi:10.1038/s41421-022-00413-2)
Supplement: Supplementary file 1 — Supplementary Figures [file 41421_2022_413_MOESM1_ESM.pdf]

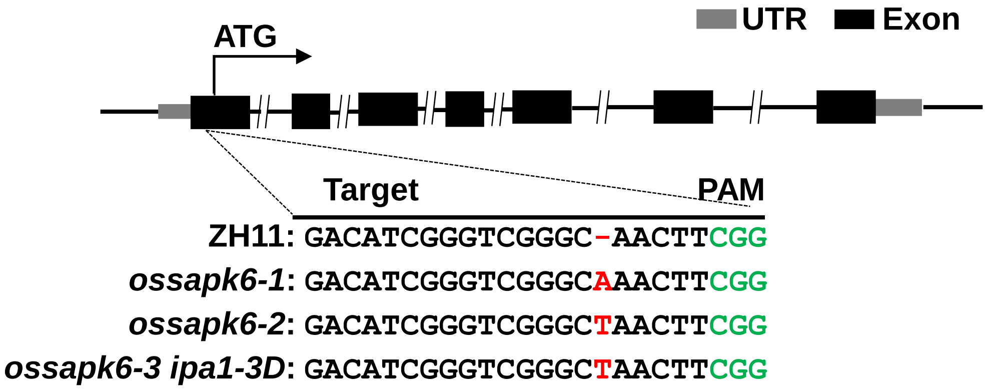

Supplementary Fig. S1 The mutation sites of *OsSAPK6* in *ossapk6-1, ossapk6-2,* and *ossapk6-3 ipa1-3D* mutants generated by CRISPR/Cas9. Related to Fig. 1.

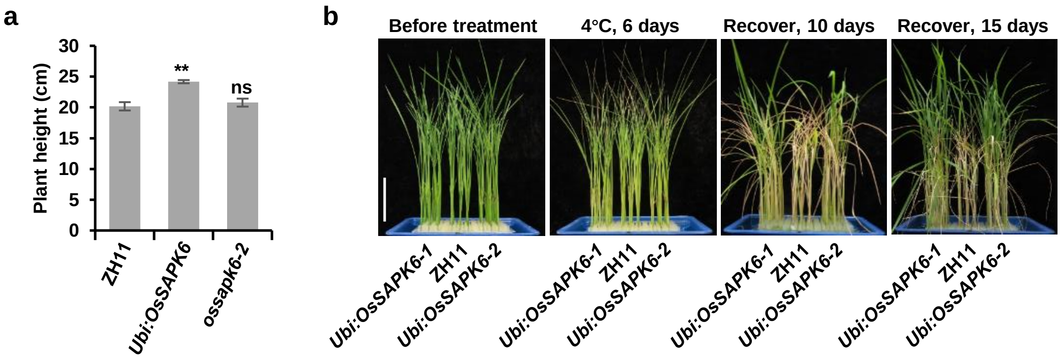

Supplementary Fig. S2 *OsSAPK6* positively regulates chilling tolerance in rice. Related to Fig. 1.

a The plant height of two-week-old wild-type ZH11, *Ubi:OsSAPK6*, *ossapk6-2* seedlings. Values are means ± SD (n = 3 biological replicates), and the asterisks indicate significant differences compared with the ZH11 (**P < 0.01; ns, no significant differences; Student's t-test). **b** Plant morphologies of two-week-old ZH11, *Ubi:OsSAPK6-1*, *Ubi:OsSAPK6-2* seedlings before treatment, after 4°C treatment for 6 days, and subsequent recovery for 10 or 15 days. Bars = 5 cm.

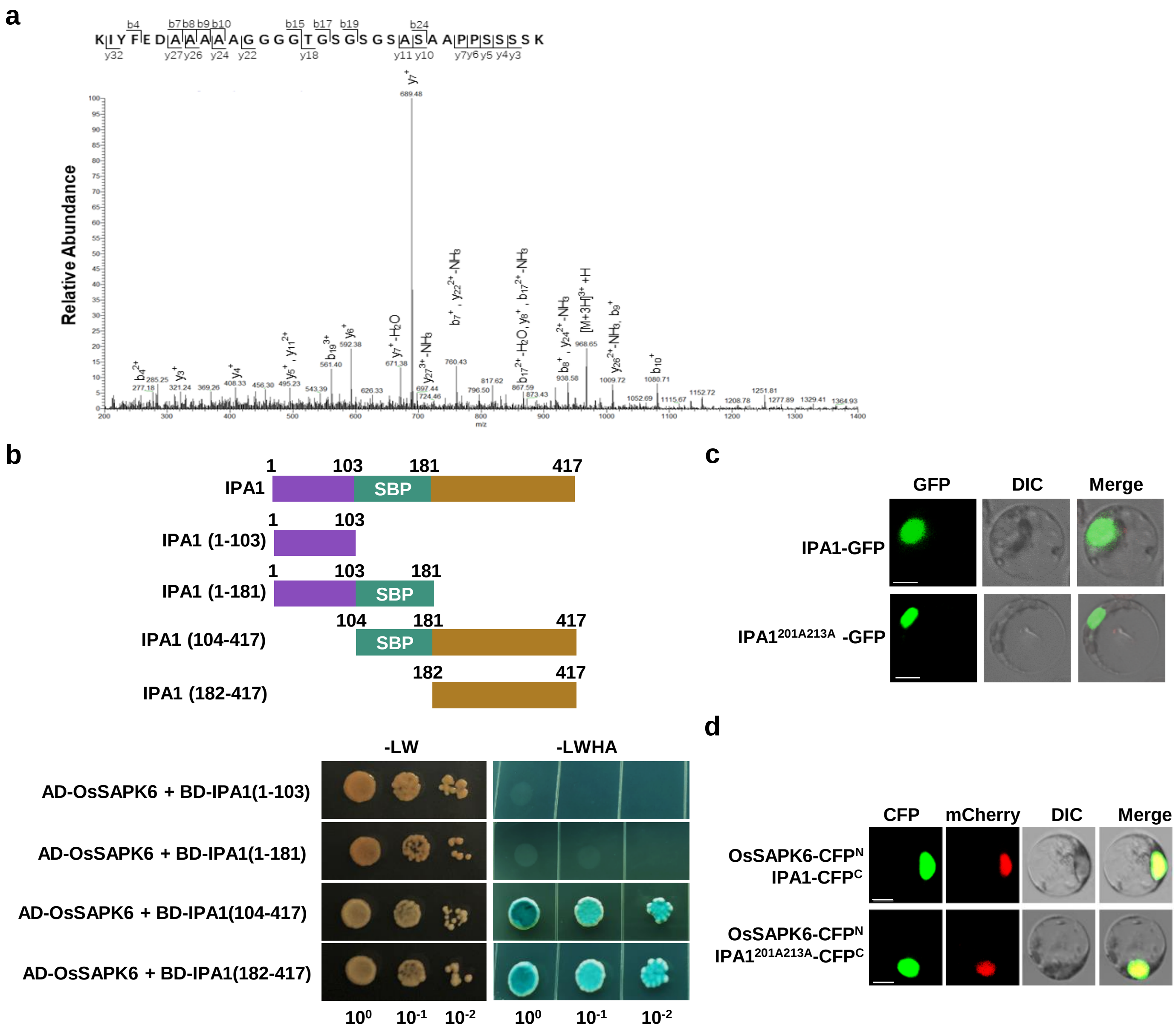

Supplementary Fig. S3 OsSAPK6 could physically interact with IPA1. Related to Fig. 2.

a IPA1 was identified as an OsSAPK6 interacting protein by LC-MS/MS analysis. **b** Yeast two-hybrid assay demonstrating the interaction between IPA1 (182-417aa) and OsSAPK6. **c** Subcellular localization of IPA1, IPA1^{201A213A} and OsSAPK6 in rice protoplasts. Scale bars, 5 μm. **d** BiFC showing the interaction between IPA1, IPA1^{201A213A} and OsSAPK6 in rice protoplasts. Scale bars, 5 μm.

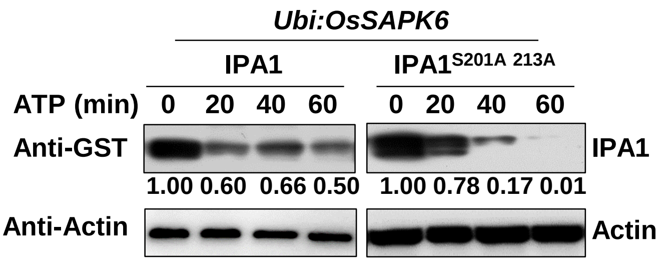

Supplementary Fig. S4 The protein degradation of IPA1 and IPA1$^{S201A\ S213A}$ in the extracts from *Ubi:OsSAPK6* plants. Related to Fig. 3.

In vitro cell-free degradation assay showing degradation of GST-IPA1 and GST-IPA1^{S201A213A} in the extracts from *Ubi:OsSAPK6* plants in the presence of ATP. GST-IPA1 was detected with anti-GST monoclonal antibody and quantitated by densitometry with actin as a control.

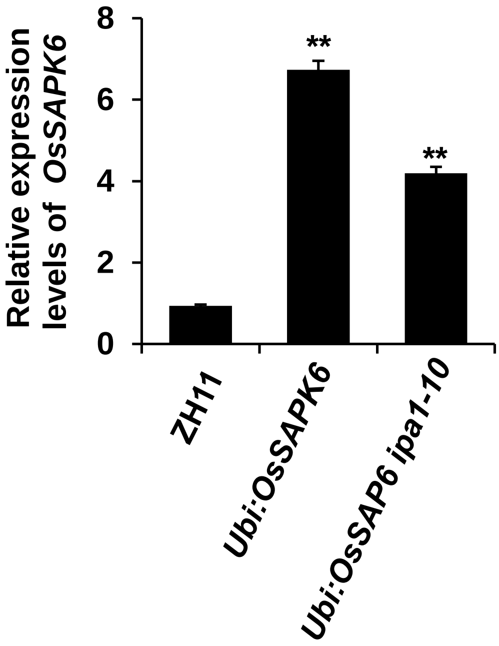

Supplementary Fig. S5 Expression levels of *OsSAPK6* in ZH11, *Ubi:OsSAPK6,* and *Ubi:OsSAPK6 ipa1-10* plants under 4°C for 6 h. Related to Fig. 4.

Values are means ± SD (n = 3 biological replicates) and the asterisks indicate significant differences compared with the ZH11 (**$P < 0.01$, Student's t-test).

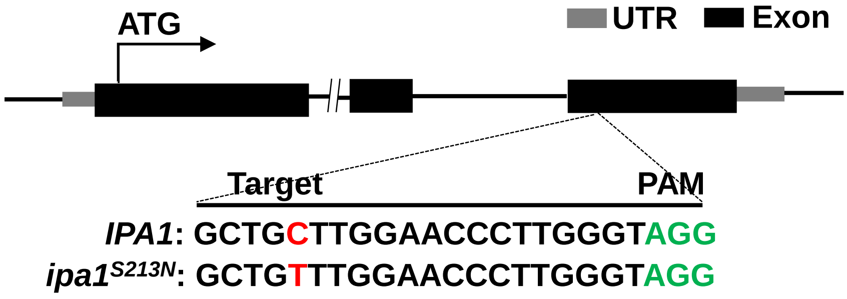

Supplementary Fig. S6 The mutation site of *IPA1* in *ipa1*^{S213N} mutants generated by CRISPR/Cas9. Related to Fig. 5.

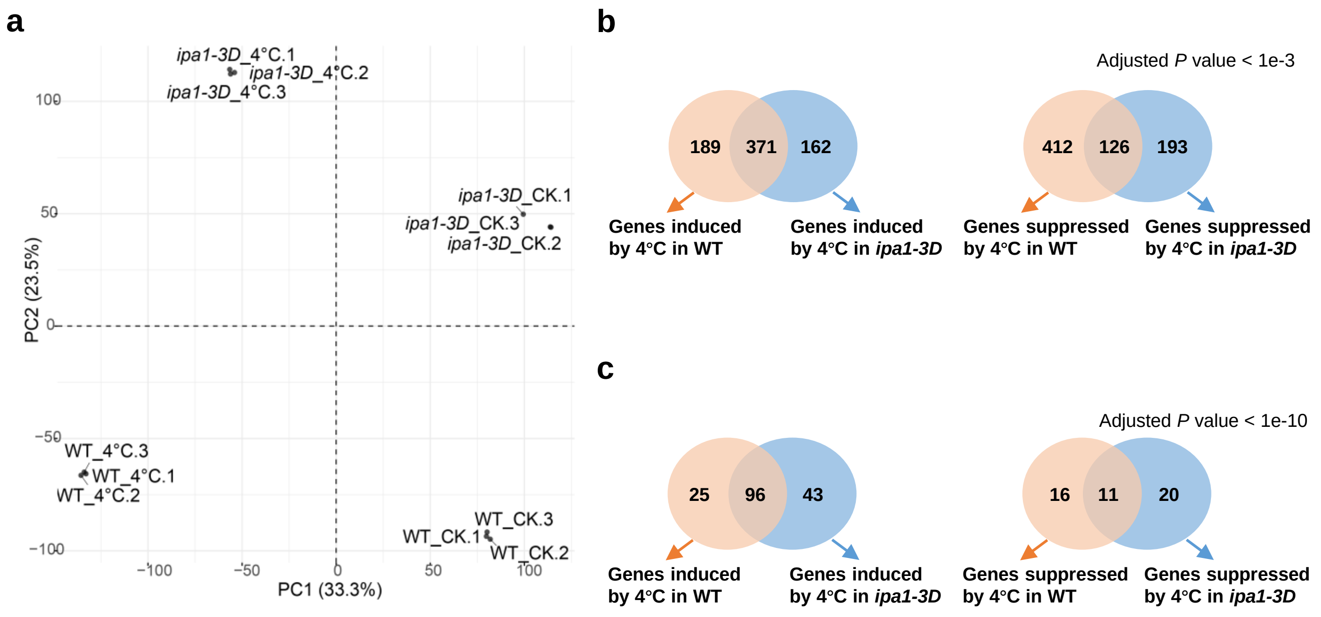

d

Gene_ID	Gene name	Description	Fold change of expression level after 6h 4°C treatment	
			WT	*ipa1-3D*
Os06g0127100	*OsCBF2*	Dehydration-responsive element-binding protein 1C.	74.71	439.56
Os08g0474000	*OsERF104*	Similar to AP2 domain containing protein RAP2.6 (Fragment).	19.65	110.28
Os03g0734500		Conserved hypothetical protein.	26.62	97.65
Os09g0457900	*OsERF102*	AP2/ERF transcription factor	799.63	2861.40
Os09g0522200	*OsCBF3*	Transcription activator	69.26	240.83
Os03g0820400	*OsZFP15*	Similar to ZPT2-13.	33.79	100.84
Os07g0225300	*OsNAC3*	OsNAC3 protein.	13.30	35.89
Os06g0133400		Conserved hypothetical protein.	22.16	55.98
Os12g0472300		Conserved hypothetical protein.	44.56	112.01
Os05g0545400	*OsMAP3K.19*	Serine/threonine protein kinase domain containing protein.	34.78	79.68
Os01g0278000	*OsVQ1*	VQ domain containing protein.	21.70	49.12
Os01g0699600	*OsMKKK62*	Mitogen-activated protein kinase kinase kinase (MAPKKK)	75.07	169.19
Os11g0299300		Lipase	131.82	268.13

Supplementary Fig. S7 Analysis of cold responsive genes in WT and *ipa1-3D*. Related to Fig. 6.

a Principal component analysis (PCA) of RNA-seq data. **b** Venn diagram showing cold-induced and -suppressed genes in WT and *ipa1-3D* (Adjusted *P* value < 1e-3). **c** Venn diagram showing cold-induced and -suppressed genes in WT and *ipa1-3D* (Adjusted *P* value < 1e-10). **d** 13 chilling-induced genes whose expression levels were greatly enhanced under chilling treatment in *ipa1-3D* compared to WT. Cutoff: $(FPKM_{ipa1\text{-}3D\_4°C}/FPKM_{ipa1\text{-}3D\_CK})/(FPKM_{WT\_4°C}/FPKM_{WT\_CK}) > 2$.

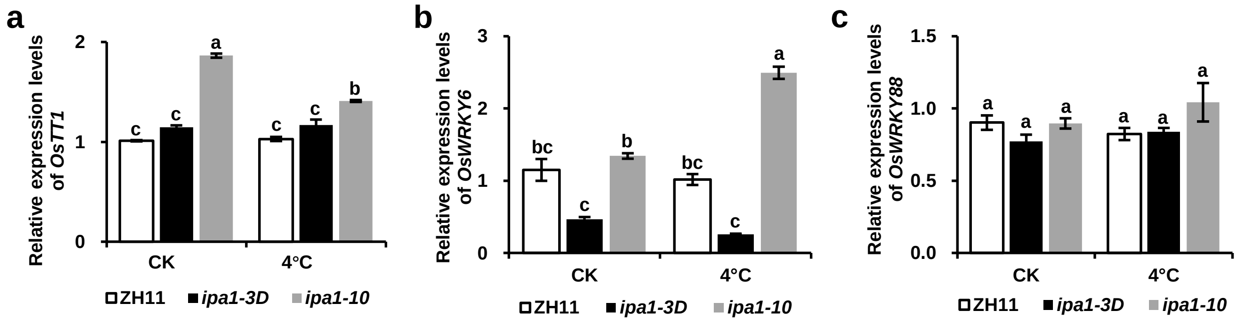

Supplementary Fig. S8 Expression levels of random selected genes in two-week-old ZH11, *ipa1-3D*, and *ipa1-10* seedlings with or without 6h 4°C treatment. Related to Fig. 6.

a-c The expression levels of *OsTT1* (**a**), *OsWRKY6* (**b**) and *OsWRKY88* (**c**) in two-week-old ZH11, *ipa1-3D*, and *ipa1-10* seedlings with or without 6h 4°C treatment. In **a**-**c**, values are means ± SD (*n* = 3 biological replicates), and the different letters indicate significant differences ($P < 0.01$) according to Tukey's honest significant difference (HSD) test.

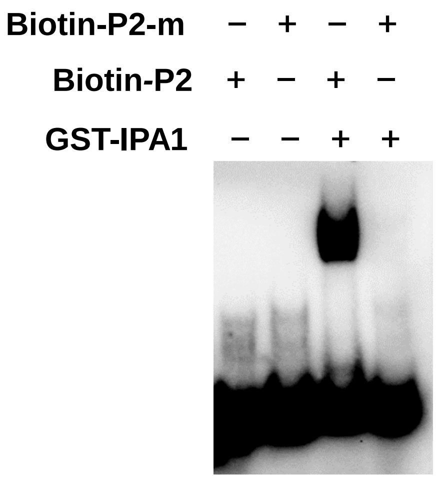

Supplementary Fig. S9 Direct binding of IPA1 to the *OsCBF3* promoter in the EMSA assay. Related to Fig. 6.

Biotin-labeled 42-bp fragment of *OsCBF3* promoter (P2) and P2 with the SBP binding motif GTAC changed to ATAC were incubated with GST-IPA1 protein.

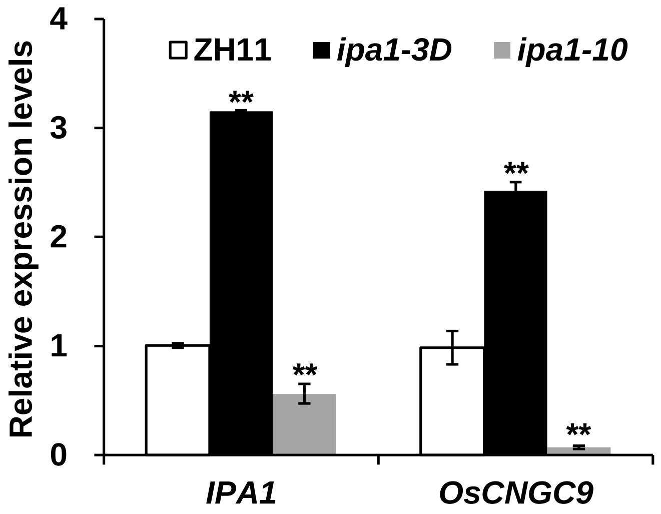

Supplementary Fig. S10 Expression levels of *IPA1* and *OsCNGC9* in two-week-old ZH11, *ipa1-3D,* and *ipa1-10* seedlings under 4°C for 6 h. Related to Fig. 6.

Values are means ± SD (n = 3 biological replicates), and the asterisks indicate significant differences compared with the ZH11 (**$P < 0.01$, Student's t-test).

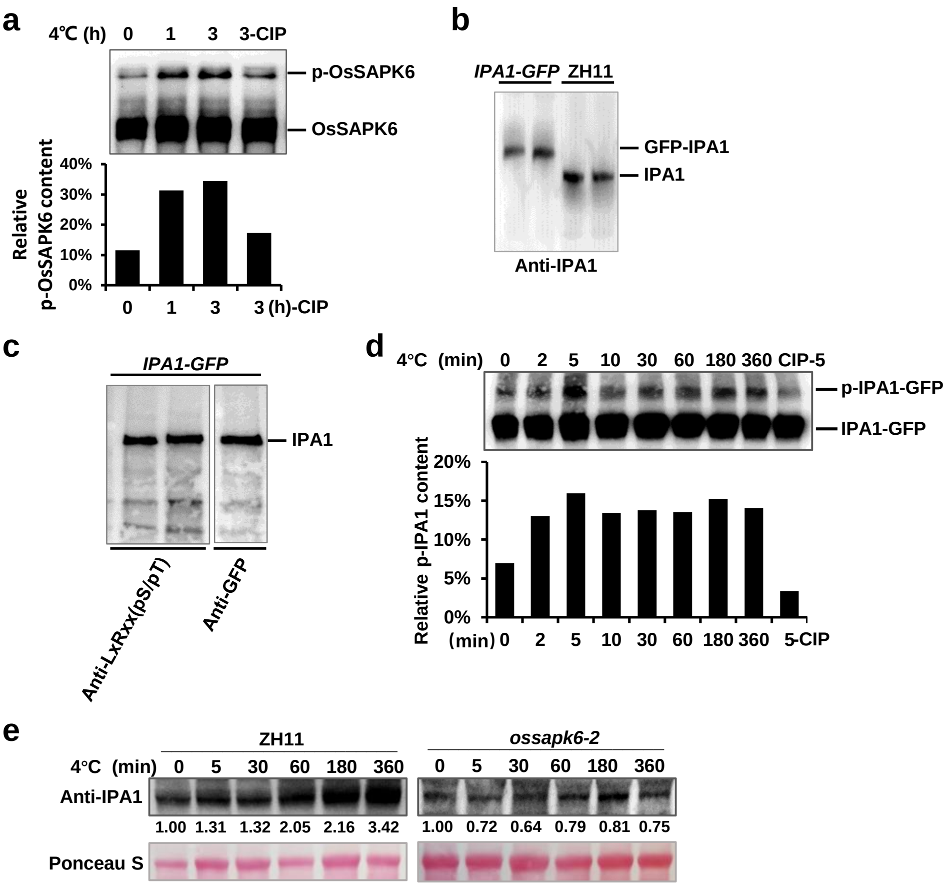

Supplementary Fig. S11 OsSAPK6 phosphorylates IPA1 under chilling stress. Related to Fig. 7.

a The autophosphorylation of OsSAPK6 was induced under chilling stress. *Flag-OsSAPK6*-OE transgenic seedlings were treated under 4°C for indicated times, and Flag-OsSAPK6 protein was then purified with anti-Flag beads, and separated in a Phos-tag gel. The phosphorylated and nonphosphorylated OsSAPK6 proteins were detected with anti-Flag antibody and quantitated by densitometry. **b** Detection of the anti-IPA1 antibody specificity using total protein extracted from *IPA1-GFP* transgenic and ZH11 seedlings. **c** Detection of the polyclonal antibody specificity against a substrate motif [LXRXX (pS/pT)] using purified IPA1-GFP protein from *IPA1-GFP* transgenic seedlings. **d** IPA1 phosphorylation profile in *ProIPA1:7mIPA1-GFP* transgenic seedlings upon 4°C treatment. Phosphorylated and nonphosphorylated IPA1 proteins were separated in a Phos-tag gel, detected with anti-GFP antibody (upper panel) and quantitated by densitometry (lower panel). **e** IPA1 protein levels in ZH11 and *ossapk6-2* seedlings under chilling stress. IPA1 protein was detected by anti-IPA1 antibody. Relative amounts of proteins were determined by densitometry and normalized to loadings determined by Ponceau staining (red).

a

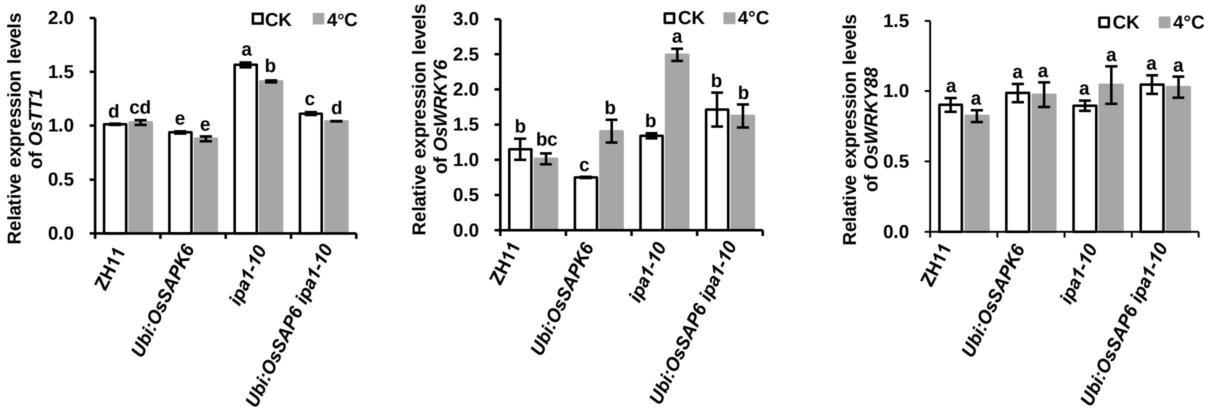

b

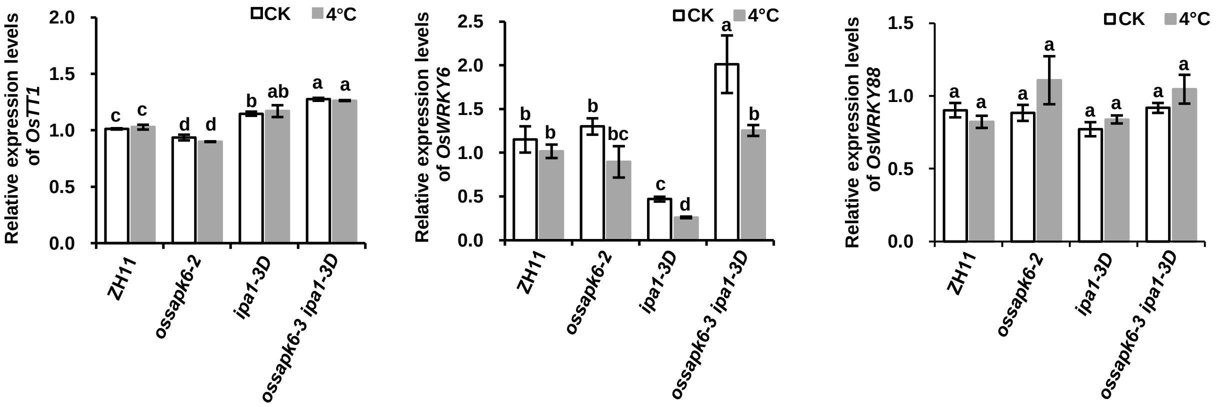

Supplementary Fig. S12 The expression level of *OsTT1*, *OsWRKY6* and *OsWRKY88* in two-week-old ZH11, *IPA1*- and *OsSAPK6*-related materials. Related to Fig. 7.

a Expression levels of *OsTT1*, *OsWRKY6* and *OsWRKY88* genes in ZH11, *Ubi:OsSAPK6*, *ipa1-10* and *Ubi:OsSAPK6 ipa1-10* with or without 6h 4°C treatment. **b** Expression levels of *OsTT1*, *OsWRKY6* and *OsWRKY88* in ZH11, *ossapk6-2*, *ipa1-3D* and *ossapk6-3 ipa1-3D* with or without 6h 4°C treatment. In **a** and **b**, values are means ± SD (n = 3 technical replicates). Different letters indicate significant differences ($P < 0.01$) according to Tukey's HSD test.

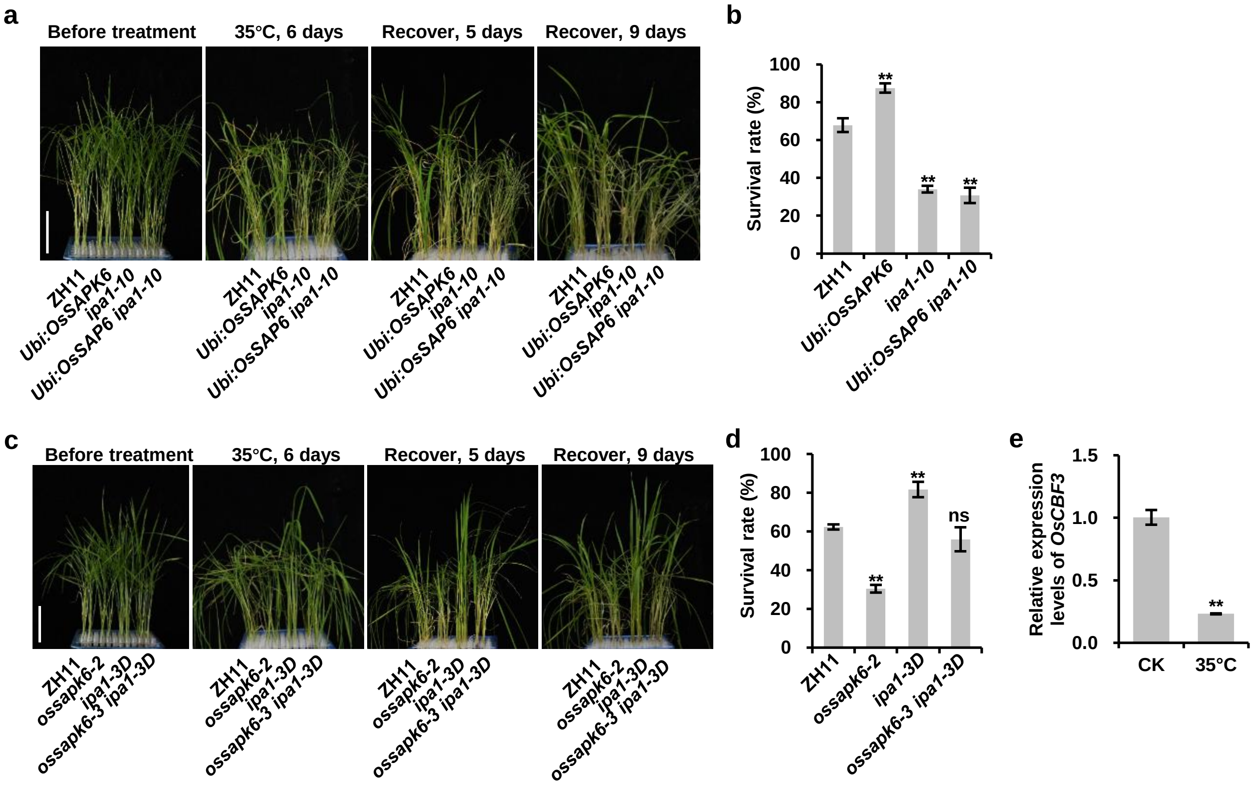

Supplementary Fig. S13 ***OsSAPK6* and *ipa1-3D* positively regulates heat tolerance in rice.** Related to Fig. 7. **a** Plant morphologies of wild-type ZH11, *Ubi:OsSAPK6*, *ipa1-10* and *Ubi:OsSAPK6 ipa1-10* seedlings before treatment, after 35°C treatment for 6 days, and subsequent recovery for 5 or 9 days. Bars = 5 cm. **b** Survival rates of wild-type ZH11, *Ubi:OsSAPK6*, *ipa1-10* and *Ubi:OsSAPK6 ipa1-10* after 35°C treatment in **a**. **c** Plant morphologies of wild-type ZH11, *ossapk6-2*, *ipa1-3D* and *ossapk6-3 ipa1-3D* seedlings before treatment, after 35°C treatment for 6 days, and subsequent recovery for 5 or 9 days. Bars = 5 cm. **d** Survival rates of ZH11, *ossapk6-2*, *ipa1-3D* and *ossapk6-3 ipa1-3D* after 35°C treatment in **c**. **e** *OsCBF3* gene expression in ZH11 with or without 35°C treatment. In **b**, **d** and **e**, values are means ± SD (n = 3 biological replicates), and the asterisks indicate significant differences compared with ZH11 in **b** and **d**, CK in **e** (**P < 0.01; ns, no significant differences; Student's t-test).
