# Supplementary material for: Chilling-induced phosphorylation of IPA1 by OsSAPK6 activates chilling tolerance responses in rice
Source: Cell Discov. 2022 Jul 26;8:71. doi: 10.1038/s41421-022-00413-2 (PMC9325753; doi:10.1038/s41421-022-00413-2)
Supplement: Supplementary file 4 — Supplementary Table 3 [file 41421_2022_413_MOESM4_ESM.docx]

**Supplemental Table 3. List of primer sequences used in this study**

| **Primer name** | **Primer Sequence** |  |
| --- | --- | --- |
| **Primers for quantitative real-time PCR** | |  |
| OsCBF1-F  OsCBF1-R  OsCBF2-F  OsCBF2-R  OsCBF3-F  OsCBF3-R | GGACCAAGTTCAGGGAGACG  GGAGTCGGCGAAGTTGAGG  GGACCAAGTTCAGGGAGACG  CCAGATGCGGGACTTCTTGT  ATCAAGCAGGAGATGAGCGG  GTCTCCCTGAACTTGGTCCG |  |
| OsCNGC9-F | GCTCTTCGTCATACGCGGTA |  |
| OsCNGC9-R | TCGAAAGCGGGAAATGGACA |  |
| IPA1-F | TGCATTCCAAGGCTCCCCGC |  |
| IPA1-R | TGCGGCAGCTGCGTTTTCCT |  |
| OsTT1-F | CGACAGCCAGTACTCCTTCT |  |
| OsTT1-R | CAACTTCTTCTCGGTGGCAA |  |
| OsWRKY6-F | GCCGCCTCCATTTCAGTAGT |  |
| OsWRKY6-R | AAAGCTCTTCATCGGCTGCT |  |
| OsWRKY88-F | CACGGGCACTTCAGTCAGAT |  |
| OsWRKY88-R | ATCTTTTGACCTGGGGCTGG |  |
| OsACTIN-F | CTTCATAGGAATGGAAGCTGCGGGTA |  |
| OsACTIN-R  P1-F (ChIP)  P1-R (ChIP)  ProUbiquitin-F (ChIP)  ProUbiquitin-R (ChIP) | CGACCACCTTGATCTTCATGCTGCTA  ATACCGAATTAGGATGTTTAAACTTGT TTCGAGCTTGTTTAGTTTGATACCT  TCGGAGACCGTGCTAGGTTT  GCCAGCGCCCATCGATT |  |
| **Primers for constructs in plant transformation** | |  |
| pJL1460-OsCBF3-F  pJL1460-OsCBF3-R | cgactctagaggatccATGTGCGGGATCAAGCAGGAGATG  gctctctagaactagtCTAGTAGCTCCAGAGTGGGACGTC |  |
| pJL1460-OsSAPK6-F | cgactctagaggatccATGGAGAAGTACGAGCTGC |  |
| pJL1460-OsSAPK6-R  gRNA(*sapk6*)  gRNA(*ipa1^S213N^*) | gctctctagaactagtTTAGAGAGGCTGAAGTGGG  GACATCGGGTCGGGCAACTTCGG  GCTGCTTGGAACCCTTGGGTAGG |  |
| **Primers for localization and BiFC** | | |
| IPA1-S201A 213A-F  IPA1-S201A 213A-R  IPA1-S201D 213D-F  IPA1-S201D 213D-R  pBI221-IPA1-GFP-F  pBI221-IPA1-GFP-R  pBI221-OsSAPK6-GFP-F  pBI221-OsSAPK6-GFP-R  pSCYCE-IPA1-F  pSCYCE-IPA1-R | GTGAGCATCGCAGGTTCAGAGCCTTTACGTTGGATTTCTCCTACCCAAGGGTTCCAGCCAGCGTAAG  GGCTGGAACCCTTGGGTAGGAGAAATCCAACGTAAAGGCTCTGAACCTGCGATGCTCAC  GTGAGCATCGCAGGTTCAGAGACTTTACGTTGGATTTCTCCTACCCAAGGGTTCCAGACAGCGTAAG  GTCTGGAACCCTTGGGTAGGAGAAATCCAACGTAAAGTCTCTGAACCTGCGATGCTCAC  agaacacgggggactctagaggatccATGGAGATGGCCAGTGGAGGA  tcctcgcccttgctcaccataagcttCAGAGACCAATCCATCGTGTTGCT  agaacacgggggactctagaggatccATGGAGAAGTACGAGCTGC  tcctcgcccttgctcaccataagcttTTAGAGAGGCTGAAGTGGG  gagagaacacgggggactctagaATGGAGATGGCCAGTGGAGGAGG  tacatcccgggagcggtaccCAGAGACCAATCCATCGTGT | |
| pSCYNE-OsSAPK6-F  pSCYNE-OsSAPK6-R | gagagaacacgggggactctagaATGGAGAAGTACGAGCTGCTCAAG  tccatcccgggagcggtaccGAGAGGCTGAAGTGGGTGAA |  |
| pSCYCE-SPL2-F | gagagaacacgggggactctagaATGGATTGGGACGCCAAGAT |  |
| pSCYCE-SPL2-R | tacatcccgggagcggtaccCCACGATGAGAAAGGAAGAGC |  |
| pSCYCE-SPL3-F | gagagaacacgggggactctagaATGGGTTCTTTTGGGATGGACT |  |
| pSCYCE-SPL3-R | tacatcccgggagcggtaccGTTCATCTGATCATAGTGGGAGT |  |
| pSCYCE-SPL6-F | gagagaacacgggggactctagaATGGAGGCTGCCCGGGTC |  |
| pSCYCE-SPL6-R | tacatcccgggagcggtaccCATTGGTCCACGTTCTAACAGCTC |  |
| pSCYCE-SPL7-F | gagagaacacgggggactctagaATGGAAGGAAACGGCTGCG |  |
| pSCYCE-SPL7-R | tacatcccgggagcggtaccGACCACGCGGGCGCCCTCCATG |  |
| pSCYCE-SPL8-F | gagagaacacgggggactctagaATGATGAACGTTCCATCCGCC |  |
| pSCYCE-SPL8-R | tacatcccgggagcggtaccGTGATCGAAGTCGAGATCAAACATC |  |
| **Primers for Y2H assays** |  |  |
| pGBDT7-IPA1-F  pGBDT7-IPA1-R  pGBDT7-IPA1(1-103aa)-F  pGBDT7-IPA1(1-103aa)-R  pGBDT7-IPA1(1-181aa)-F  pGBDT7-IPA1(1-181aa)-R  pGBDT7-IPA1(104-417aa)-F  pGBDT7-IPA1(104-417aa)-R  pGBDT7-IPA1(182-417aa)-F  pGBDT7-IPA1(182-417aa)-R  pGADT7-OsSAPK6-F  pGADT7-OsSAPK6-R | catggaggccgaattcATGGAGATGGCCAGTGGAGG  cagctcgagctcgatggatccCAGAGACCAATCCATCGTGTTG  catggaggccgaattcATGGAGATGGCCAGTGGAGG  aggtcgacggatccCCGCGGCGGCGGCGGCGG  catggaggccgaattcATGGAGATGGCCAGTGGAGG  aggtcgacggatccAGGGGTTTGCGGCCTCCTC  catggaggccgaattcATGTGCCAGGTGGAGGGG  aggtcgacggatccCTACAGAGACCAATCCATCGTGTT  catggaggccgaattcATGTTGGCATCACGCTACGG  aggtcgacggatccCTACAGAGACCAATCCATCGTGTT  catatggccatggaggccagtgaattcATGGAGAAGTACGAGCTGCTCAAG  atctgcagctcgagctcgatggatccGAGAGGCTGAAGTGGGTGAAT |  |
| **Primers for protein expression** | |  |
| pGEX4t-1-IPA1-F  pGEX4t-1-IPA1-R | ctgttccaggggcccctgggatccGAGATGGCCAGTGGAGGAGG  ccgctcgagtcgacccgggaattcCTACAGAGACCAATCCATCGTGT |  |
| pET28a(+)-OsSAPK6-F | gcaaat gggtcgcggatccGAGAAGTACGAGCTGCTCAAG |  |
| pET28a(+)-OsSAPK6-R | gtcgacggag ctcgaattcGAGAGGCTGAAGTGGGTGAA |  |
| **Probes for EMSA assays** |  |  |
| P2-F  P2-R | Bio-TACCTAGGTTTTAAGTTATA**GTAC**TTACAATTTTTTGATGAG  CTCATCAAAAAATTGTAAGTACTATAACTTAAAACCTAGGTA |  |
| P2-Cold-F | TACCTAGGTTTTAAGTTATA**GTAC**TTACAATTTTTTGATGAG |  |
| P2-mutant-F  P2-mutant-R | Bio-TACCTAGGTTTTAAGTTATA**ATAC**TTACAATTTTTTGATGAG  CTCATCAAAAAATTGTAAGTATTATAACTTAAAACCTAGGTA |  |
| **Primers for transient transactivation assays** | |  |
| pGreen0800-ProOsCBF3-F  pGreen0800-ProOsCBF3-R  pBI221-IPA1-GFP-F  pBI221-IPA1-GFP-R  pBI221-OsSAPK6-GFP-F  pBI221-OsSAPK6-GFP-R | tatagggcgaattgggtaccAATTACTCCTAACCAGCATCAATCC  tatcgataccgtcgacCTTCGGATTTGTGTGTGTGTTTCTG  agaacacgggggactctagaggatccATGGAGATGGCCAGTGGAGGA  tcctcgcccttgctcaccataagcttCAGAGACCAATCCATCGTGTTGCT  agaacacgggggactctagaggatccATGGAGAAGTACGAGCTGC  tcctcgcccttgctcaccataagcttTTAGAGAGGCTGAAGTGGG |  |
